# Single-Exposure Prophylactic Transcranial Nano-Pulsed Laser Therapy Promotes Functional Resilience Following Mild Blast-Induced Neurotrauma

**DOI:** 10.3390/ijms27167505

**Published:** 2026-08-21

**Authors:** Nikita Gupta, Katherine N. Sheffield, Mohammadhossein Khanmirzaei, Auston C. Grant, Jutatip Guptarak, Ian J. Bolding, Kathia M. Johnson, Rinat O. Esenaliev, Donald S. Prough, Maria-Adelaide Micci

**Affiliations:** 1 Department of Anesthesiology, University of Texas Medical Branch, Galveston, TX 77550, USAkasheffi@utmb.edu (K.N.S.); ian.bolding@bcm.edu (I.J.B.);; 2 John Sealy School of Medicine, University of Texas Medical Branch, Galveston, TX 77550, USA; 3 Department of Neurobiology, University of Texas Medical Branch, Galveston, TX 77550, USA; riesenal@utmb.edu; 4 Moody Brain Health Institute, University of Texas Medical Branch, Galveston, TX 77550, USA

**Keywords:** traumatic brain injury, blast-induced neurotrauma, nano-pulsed laser therapy, optoacoustic neuromodulation, preconditioning, functional resilience, BDNF, neuroinflammation

## Abstract

Blast-induced traumatic brain injury is a prevalent and underreported condition, particularly among military service members, for whom effective prophylactic interventions are lacking. Nano-pulsed laser therapy (NPLT) is a non-invasive neuromodulatory approach that delivers short pulses of near-infrared light to generate optoacoustic effects within cerebral tissue and has previously demonstrated therapeutic benefit following TBI. In this study, we evaluated whether a single pre-exposure application of NPLT could confer protection against neurological, cognitive, and cellular sequelae of mild blast injury. Adult male Sprague-Dawley rats were randomized to receive NPLT or Sham treatment 24 h prior to either Sham or mild blast exposure using the Advanced Blast Simulator. Neurological reflexes and vestibulomotor function were assessed on post-injury days (PIDs) 1–5, while cognitive performance was evaluated using the Morris Water Maze on PIDs 13–17. Histological analyses of microglia, astrocytes, and myelination were performed on PID 17. A single mild blast did not significantly alter gross neurological function but was associated with deficits in fine motor coordination and cognitive performance. Pre-exposure NPLT modestly attenuated blast-associated fine motor dysfunction, with a significant improvement compared with TBI on PID 4. In the Morris Water Maze, TBI animals exhibited significantly increased latency compared with Sham on PIDs 13 and 17, whereas NPLT + TBI animals did not significantly differ from Sham across the testing period, consistent with preservation of cognitive performance. Histological responses were regionally heterogeneous: NPLT alone produced distinct glial alterations, while NPLT + TBI animals exhibited a mixture of treatment- and injury-associated responses rather than uniform normalization to uninjured controls. NPLT did not prevent localized blast-associated reductions in corpus callosum myelin staining. In naive animals, NPLT significantly increased hippocampal brain-derived neurotrophic factor (BDNF) mRNA expression 24 h after treatment. A single pre-injury application of NPLT was associated with functional resilience following mild blast exposure despite persistent and regionally heterogeneous histopathological alterations. Increased hippocampal BDNF 24 h after NPLT, together with region-specific glial changes following NPLT in the absence of injury, demonstrates that a single treatment produces sustained molecular and cellular effects before blast exposure. These findings are consistent with the hypothesis that prophylactic NPLT establishes an altered pre-injury biological state that may modify the subsequent response to blast and support further investigation of NPLT as a prophylactic strategy and of the mechanisms underlying NPLT-associated preconditioning.

## 1. Introduction

The Center for Disease Control and Prevention (CDC) estimates there are approximately 2.5 million traumatic brain injury (TBI)-related visits to the emergency department each year in the U.S. [1]. Globally, 64–74 million people sustain TBI annually, making TBI a leading cause of death and disability in the world [2]. However, the true incidence of TBI is likely higher, because mild TBIs, also known as concussive injuries, account for 80–90% of all TBIs [1,2] and are often never reported. Moreover, because TBI is not a one-time occurrence, but rather a disease process [3], an estimated 1.1% of people, or 3.17 million people, are living with long-term TBI-related disabilities in the U.S. [4]. This percentage skyrockets to 25% among U.S. service members who experience TBI-related hospitalizations [5]. In the U.S., the financial burden of TBI is measured by the direct costs, deaths within and outside of the medical system, and costs of medical treatments of hospitalized and non-hospitalized TBI patients. The nonfatal healthcare costs are estimated to be over USD 40.6 billion in the U.S. [6]. The second most important component of the financial burden to the U.S. caused by TBI is related to reduced productivity from loss of wages and fringe benefits. One Canadian study followed approximately 18,000 patients with TBI for 3 years post-injury and estimated their total personal loss to be CAD 588 million (USD 435 million) [7]. Considering its underreported incidence rate, long-term repercussions, and financial toll, TBI continues to be a “silent epidemic”, an infamy designated more than 30 years ago [8].

The military population is especially vulnerable to TBI due to blast exposure from explosive devices or mortars. Soldiers return home from military zones with this “signature injury”, leading to a unique subset of symptoms that can cause long-term disability [9]. TBIs caused by blast exposures (blast TBI or bTBI) are characterized by a primary blast injury caused by a positive pressure wave of rapidly released energy that compresses the surrounding air and propels outward [10]. It is hypothesized that blast-induced neurotrauma (BINT) is caused by (1) acceleration and rotational forces from the shock wave directly passing through the skull as part of the wave is absorbed and another part is reflected and (2) the systemic vascular changes that occur as kinetic energy from the blast is transferred to the tissues, organs, and fluids [10]. Both have been shown to contribute to the complex, dynamic disease process that follows the primary injury, including increased membrane permeability, axonal injury, upregulation of proinflammatory cytokines, and activation of neuroimmune cells [9]. In one study, mice exposed to blast waves showed evidence of compromised cell membranes and an increase in total tau protein [11]. In a swine model, blast exposure led to axonal damage in prominent white matter tracts, astrocyte activation in the hippocampus, and evidence of increased damage to myelin [12].

Within the last decade, TBI research has seen an increase in funding [13], yet there remains a dearth of therapeutic interventions to alleviate and protect against the burden of TBI. Most current treatments are for moderate to severe TBI and can be invasive in nature, including head elevation, seizure prophylaxis, hyperventilation to treat intracranial hypertension, medically induced comatose state, and craniotomies. Mild TBI (mTBI) intervention includes monitoring for signs of a concussion, painkillers, or possible seizure prophylaxis. Overall, these treatments are supportive and do not treat the underlying molecular and cellular pathology. One promising option is a novel, patented neuromodulatory approach called nano-pulsed laser therapy (NPLT), a medical-grade transcranial optoacoustic system that delivers near-infrared light (NIL; 808 nm) in short (10 ns), low-energy (5 mJ) pulses at a repetition rate of 20 Hz [14]. These pulses cause a local rise in temperature and pressure (typically to a 3 mm delivery site), which propagates a thermoelastic response in blood. Hemoglobin, a chromophore, absorbs the light, which generates a fleeting cellular expansion; as it dissipates, the red blood cell contracts. This oscillating expansion and contraction from the NIL pulses is proposed to generate an ultrasound (US) wave through the blood [15]. We have previously shown that NPLT reduced microglial activation and improved vestibulomotor and neurocognitive outcomes in rats subjected to bTBI [14,16].

Because of this hopeful outcome of NPLT as a post-TBI treatment modality, the natural next step was to test its ability to prophylactically protect against TBI. Specifically, the aim of this experiment was to determine the efficacy of NPLT as a pre-exposure prophylactic (PrEP) treatment for bTBI. Using a rat model of mild blast exposure, we report that a single pre-injury application of NPLT preserves cognitive performance and modestly attenuates fine motor dysfunction despite persistent localized myelin abnormalities and heterogeneous glial responses. The outcome of such an experiment is significant given the large population of U.S. service members who are disproportionately vulnerable to bTBI and its potential long-term consequences.

## 2. Results

### 2.1. NPLT Increases Hippocampal BDNF Expression 24 h After Treatment in Naive Rats

**Figure 1:**
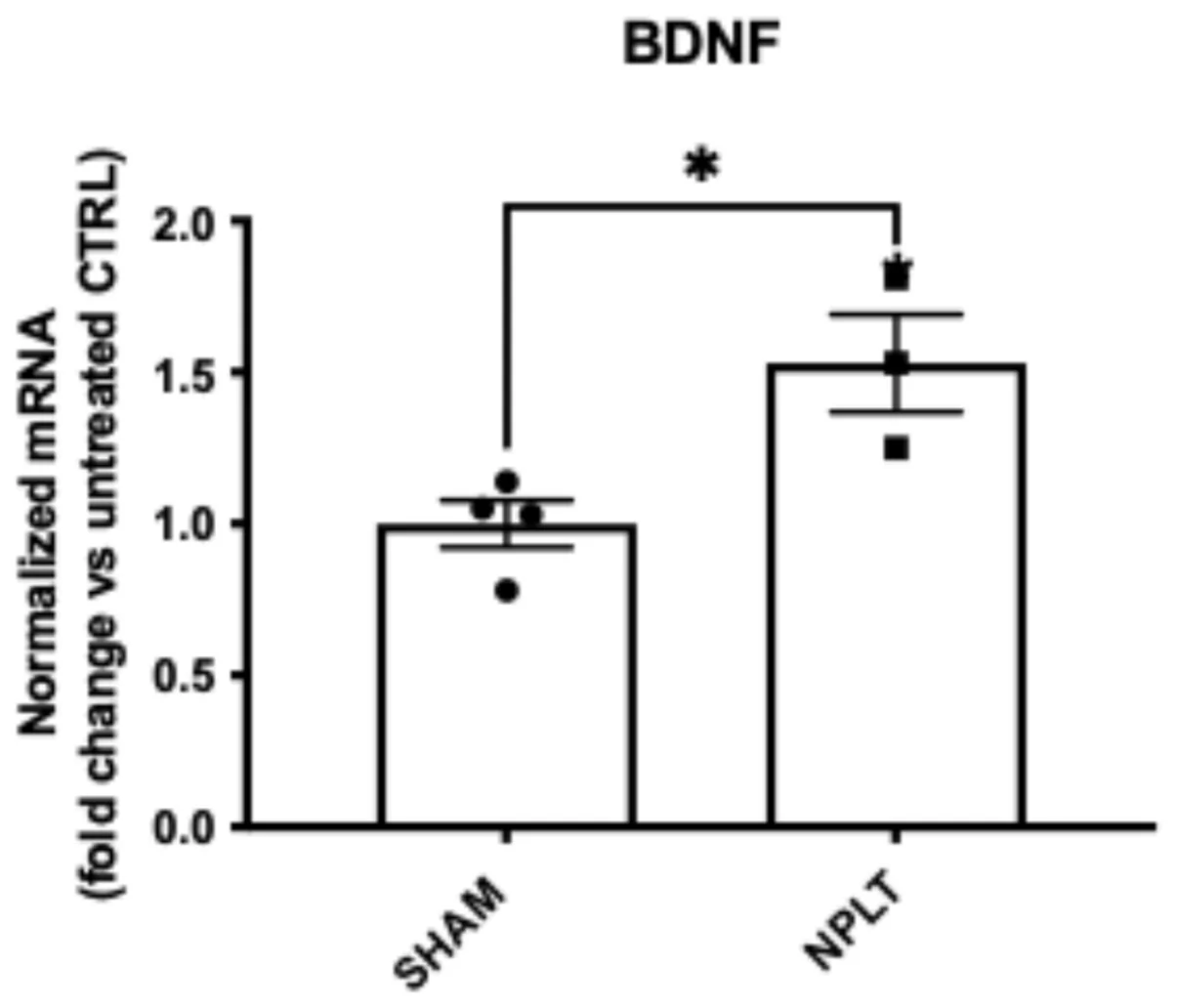
NPLT increases BDNF expression in the hippocampus of naive rats 24 h after treatment. BDNF mRNA expression was normalized to GAPDH and is presented as mean ± SEM. * *p* < 0.05, Student’s *t*-test.

To assess the molecular effects of NPLT in the absence of injury, naive rats received a bilateral, 5 min application of NPLT or Sham treatment, and were euthanized 24 h later. The expression of the neuroprotective factor BDNF was significantly increased in the hippocampus 24 h after NPLT treatment, as compared with Sham-treated rats (Figure 1).

### 2.2. Single Mild Blast Does Not Significantly Disrupt Neurological Reflexes

**Figure 2:**
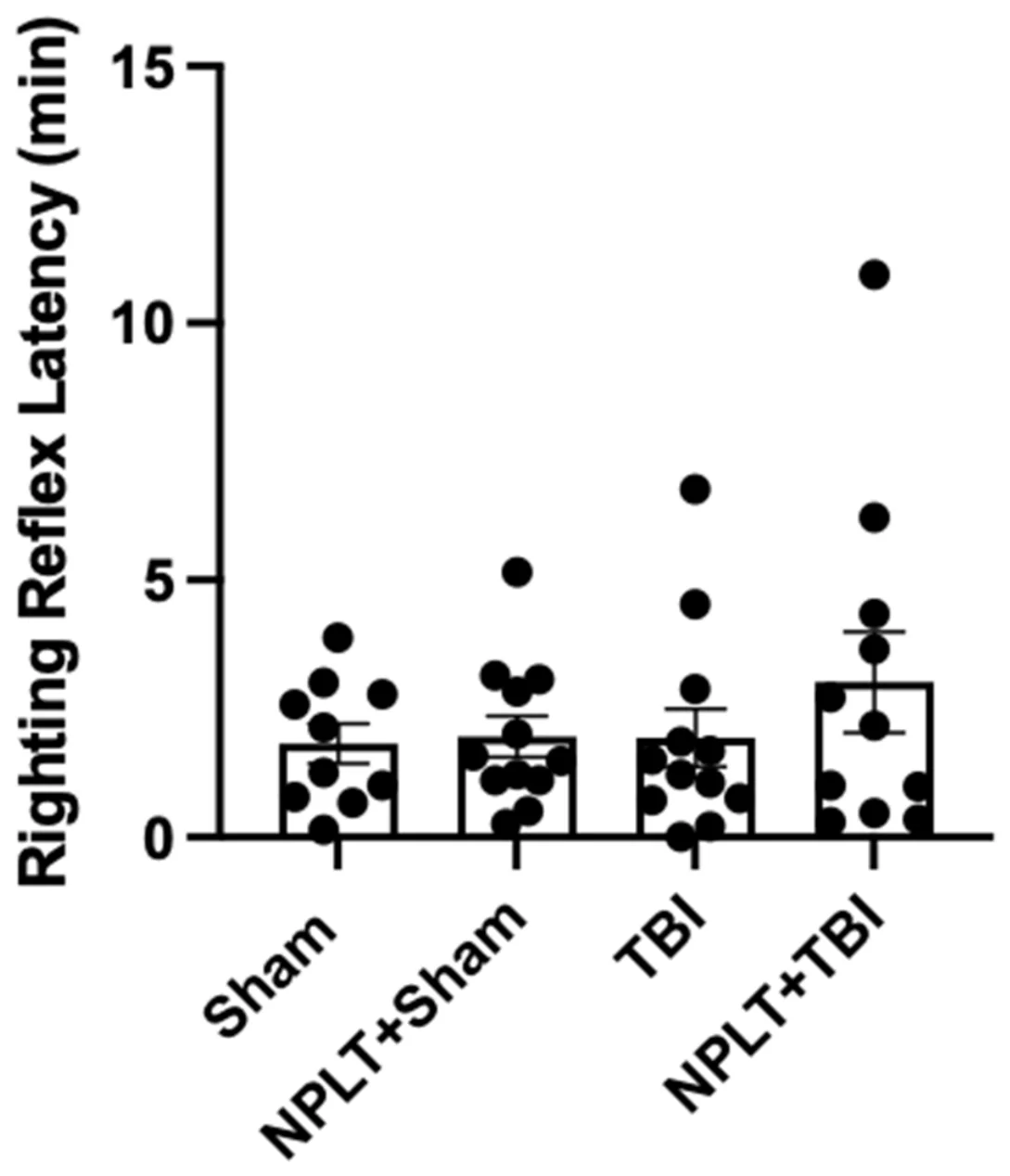
NPLT does not affect the duration of righting reflex suppression. No significant differences in the duration of righting reflex suppression were observed among the Sham, NPLT + Sham, TBI, and NPLT + TBI groups. Data is presented as mean +/− SEM. Data were analyzed using Brown–Forsythe and Welch ANOVA.

**Figure 3:**
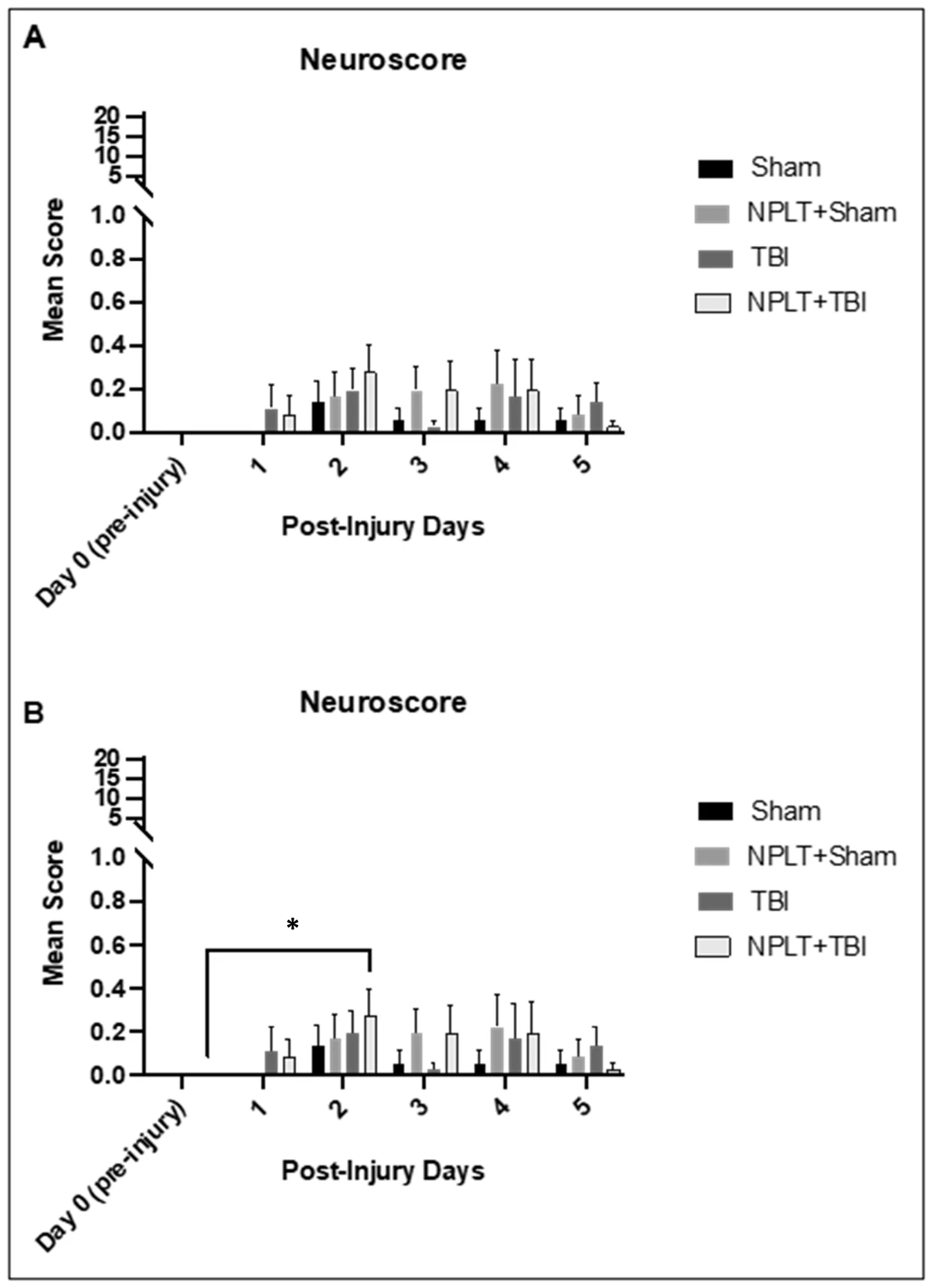
Assessment of neurological reflexes. (**A**) No significant differences in mean Neuroscore between Sham, NPLT + Sham, TBI, and NPLT + TBI on PIDs 0–5 (*n* = 12/group). (**B**) Within-group analysis shows a significant increase in mean Neuroscore in NPLT + TBI on day 0 compared to day 2 (* *p* = 0.030, two-way ANOVA). Data is presented as mean +/− SEM.

To evaluate the effect of bTBI and NPLT on neurological function, rats were subjected to reflex testing at two timepoints, the RR test immediately following the blast and the Neuroscore assessment on PIDs 1–5. Immediately following blast injury, there were no significant differences between treatment groups in the RR test (Figure 2). In the PIDs 1–5 Neuroscore assessment (Figure 3), there were no significant differences between treatment groups, while a within-group analysis revealed a significant difference (*p* = 0.030) between pre-injury baseline and PID 2 for NPLT + TBI. Although non-significant, the Neuroscore of NPLT + TBI peaked on PID 2 and then decreased over PIDs 3–5.

### 2.3. Prophylactic NPLT Variably Attenuates Fine Motor Dysfunction After Single Mild Blast

**Figure 4:**
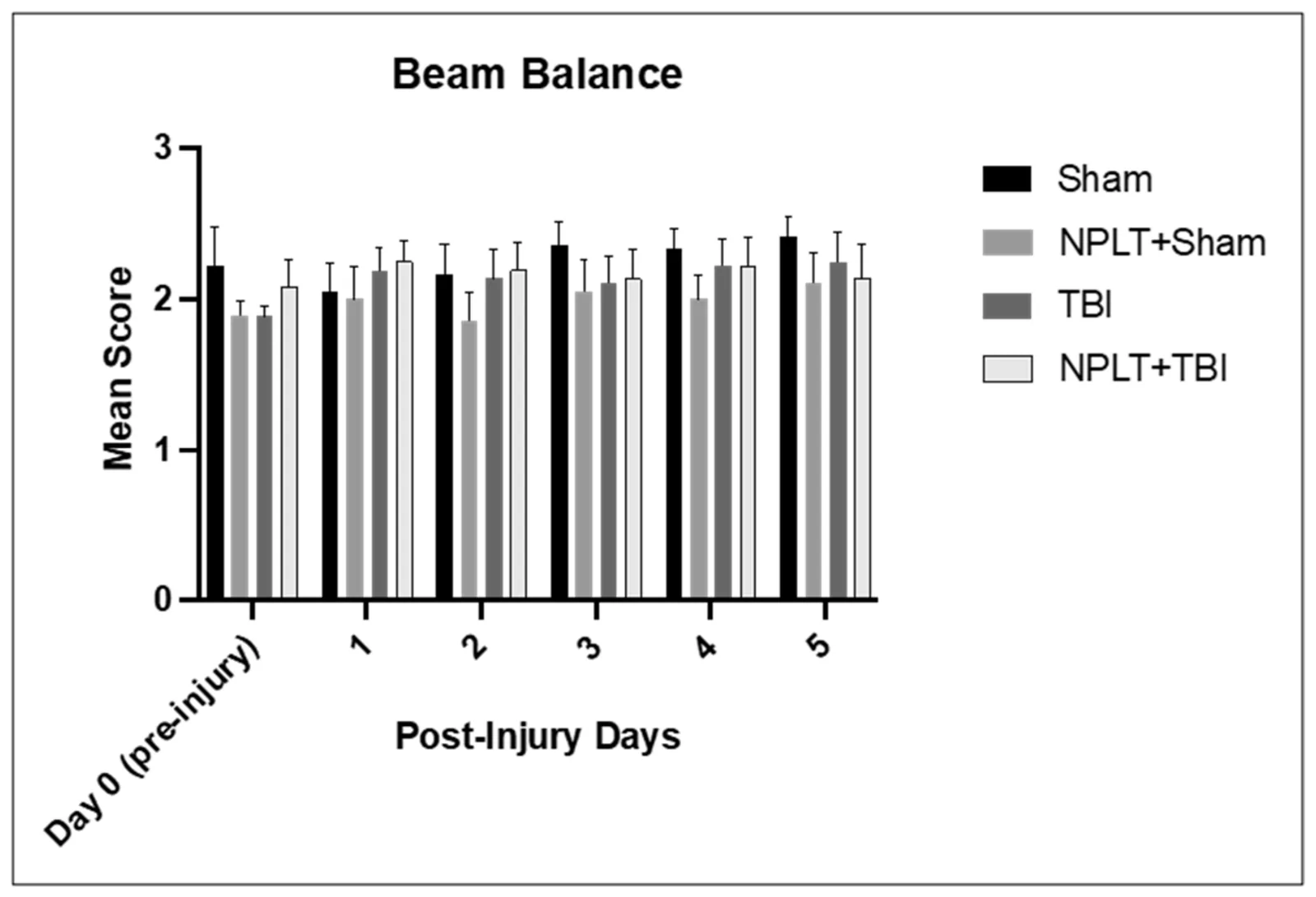
Assessment of gross vestibulomotor coordination. Beam balance scoring did not significantly differ between the Sham, NPLT + Sham, TBI, and NPLT + TBI on PIDs 0–5 (*n* = 12/group). Data is presented as mean +/− SEM.

**Figure 5:**
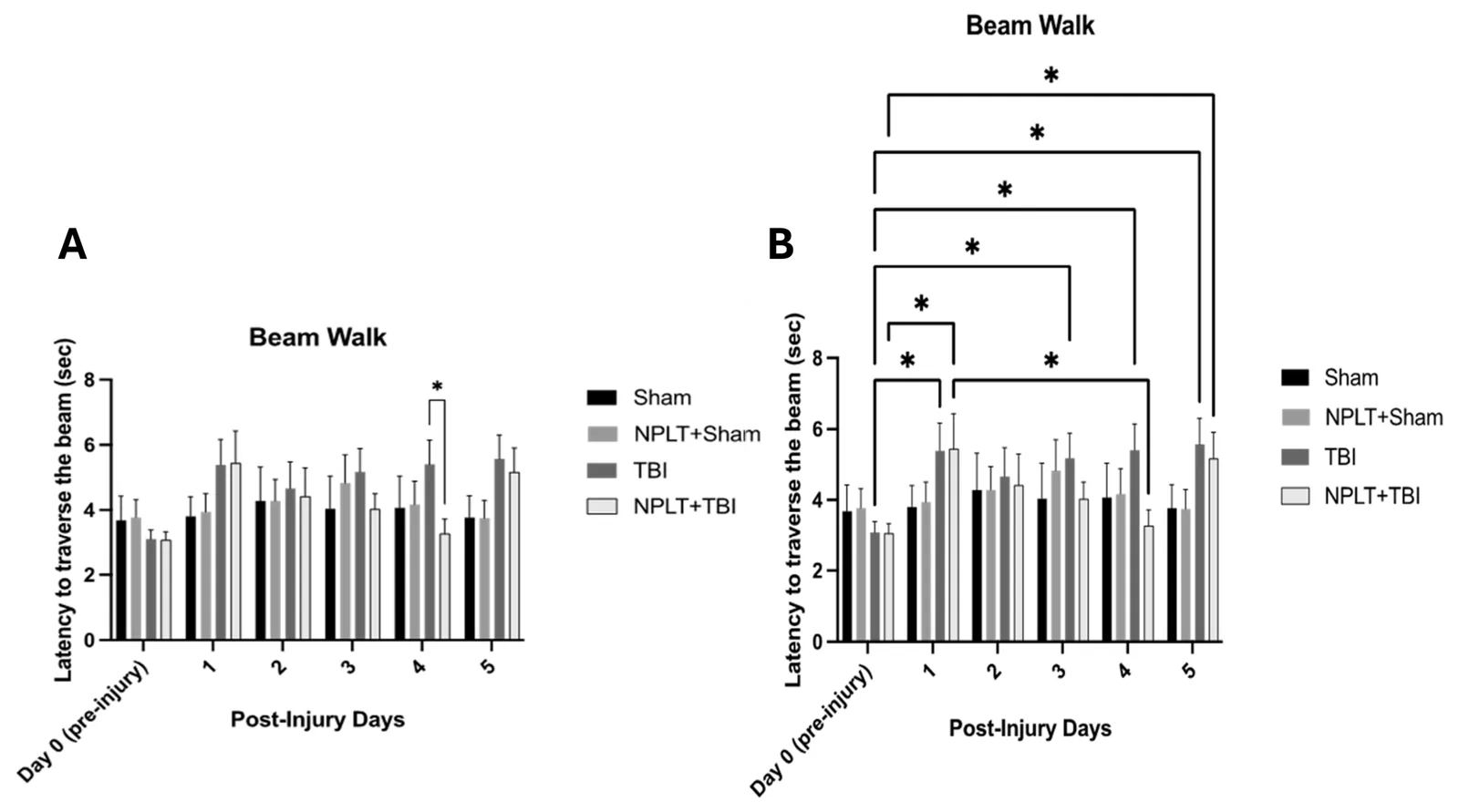
Assessment of fine motor coordination. (**A**) Between-group analysis shows a significant reduction in the latency to traverse the beam on PID 4 in NPLT + TBI when compared to TBI (*p* = 0.0408), which is accompanied by a non-significant trend in reduction in NPLT + TBI when compared to TBI on PIDs 2, 3, and 5. (**B**) Within-group analysis depicts significant differences in beam walk latency between PIDs 0 to 5. Significant differences were observed in TBI between PID 0 vs. PID 1 (*p* = 0.00266), PID 0 vs. PID 3 (*p* = 0.044), PID 0 vs. PID 4 (*p* = 0.026), and PID 0 vs. PID 5 (*p* = 0.017). Significant differences were also observed in NPLT + TBI on PID 0 vs. PID 1 (*p* = 0.022), PID 0 vs. PID 5 (*p* = 0.042), and PID 1 vs. PID 4 (*p* = 0.037). All statistical tests were performed with two-way ANOVA. Data is presented as mean +/− SEM. * *p* < 0.05.

To evaluate the effect of PrEP NPLT on gross vestibulomotor coordination, rats were assessed on the beam balance on PIDs 1–5; however, there were no significant differences between treatment groups (Figure 4). To evaluate changes to fine motor coordination, rats were subjected to the beam walk on the same days (Figure 5). There was a non-significant trend for TBI to increase the time to traverse the beam compared to Sham on PIDs 1–5 and for NPLT + TBI to decrease the time to traverse compared to TBI on PIDs 2, 3, and 5. On PID 4, there was a significant (*p* = 0.0408) reduction in time to traverse the beam in NPLT + TBI compared to TBI. Within-group analysis revealed that a single blast increased time to traverse the beam in the TBI on PIDs 1, 3, 4, and 5 (*p* = 0.00266, *p* = 0.044, *p* = 0.026, and *p* = 0.017, respectively) compared to the TBI group’s pre-injury baseline. Notably, while NPLT + TBI transiently increased on PID 1 (*p* = 0.022) and PID 5 (*p* = 0.042) compared to baseline, NPLT + TBI reduced time to traverse the beam non-significantly on PIDs 2 and 3, and significantly on PID 4 (*p* = 0.037) compared to PID 1 (Figure 5).

### 2.4. Prophylactic NPLT Prevents Disruption of Cognitive Function After Single Mild Blast

**Figure 6:**
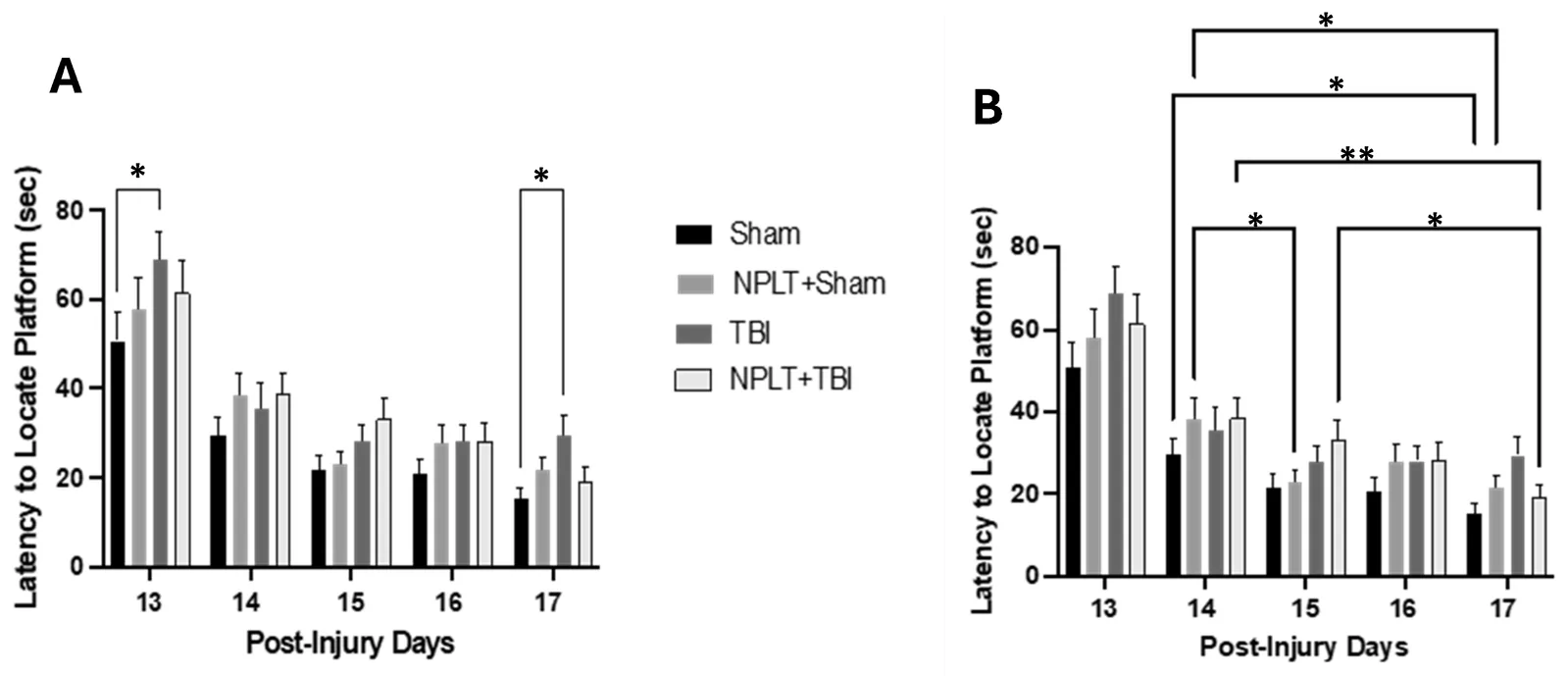
Morris Water Maze. (**A**) Between-group analysis of latency to locate the platform during trial 2 shows increased latency in TBI compared to Sham on PID 13 (*p* = 0.007) and PID 17 (*p* = 0.036). (**B**) Within-group analysis of latency (trial 2) shows decreased latency in Sham from PID 14 to 17 (*p* = 0.034), in NPLT + Sham from PID 15 to 17 (*p* = 0.023, *p* = 0.014), and in NPLT + TBI on PID 17 vs. PID 14 and 15 (*p* = 0.004, *p* = 0.039). No significant decrease is observed in TBI from PIDs 15–17 vs. PID 14. Data is presented as mean +/− SEM. All analyses were performed using two-way ANOVA. * *p* < 0.05, ** *p* < 0.01.

To assess changes in cognitive function after single blast injury and PrEP NPLT, rats were subjected to the working memory paradigm of the MWM test (Figure 6) on PIDs 13–17 in which they were evaluated for how long it took them to find a hidden platform they had been pre-trained to find (i.e., the latency period), during T1 and T2. Longer latency times indicate increased cognitive dysfunction. There was a significant increase in latency in the TBI group compared to Sham on PID 13 (*p* = 0.007). TBI latency remained increased compared to Sham non-significantly on PIDs 14, 15, and 16 and significantly on PID 17 (*p* = 0.036). Notably, NPLT + TBI was not significantly different from Sham on PIDs 13–17, indicating PrEP NPLT prevented TBI-induced cognitive dysfunction. Within-group analysis showed that all groups significantly decreased in latency across PIDs 14–17 when compared to PID 13, the first day of testing. Furthermore, on PID 17, the last day of testing, all groups were significantly decreased compared to their performance on PID 14, except the TBI group.

### 2.5. Prophylactic NPLT Differentially Modulates Microglial Responses Across Cortical and Subcortical Regions Following Mild Blast Injury

**Figure 7:**
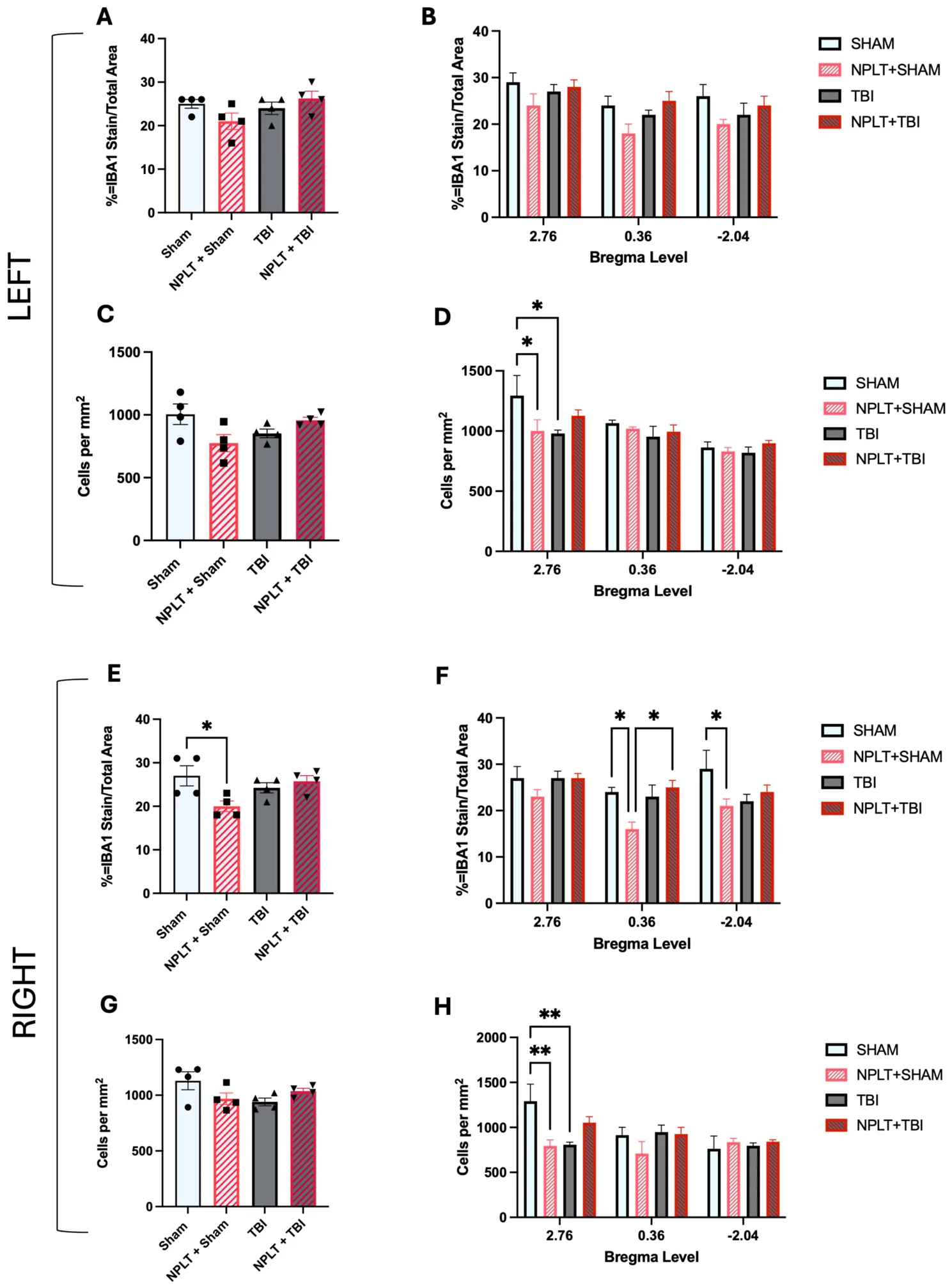
Microglia in the motor cortex. Graphs (**A**–**D**) show Iba1 percent stain (**A**,**B**) and cell count (**C**,**D**) in the left hemisphere of the motor cortex, while (**E**–**H**) show the same measures in the right hemisphere of the motor cortex. Data is presented as mean +/− SEM. * *p* < 0.05, ** *p* < 0.01 one-way ANOVA with Tukey’s post hoc test.

**Figure 8:**
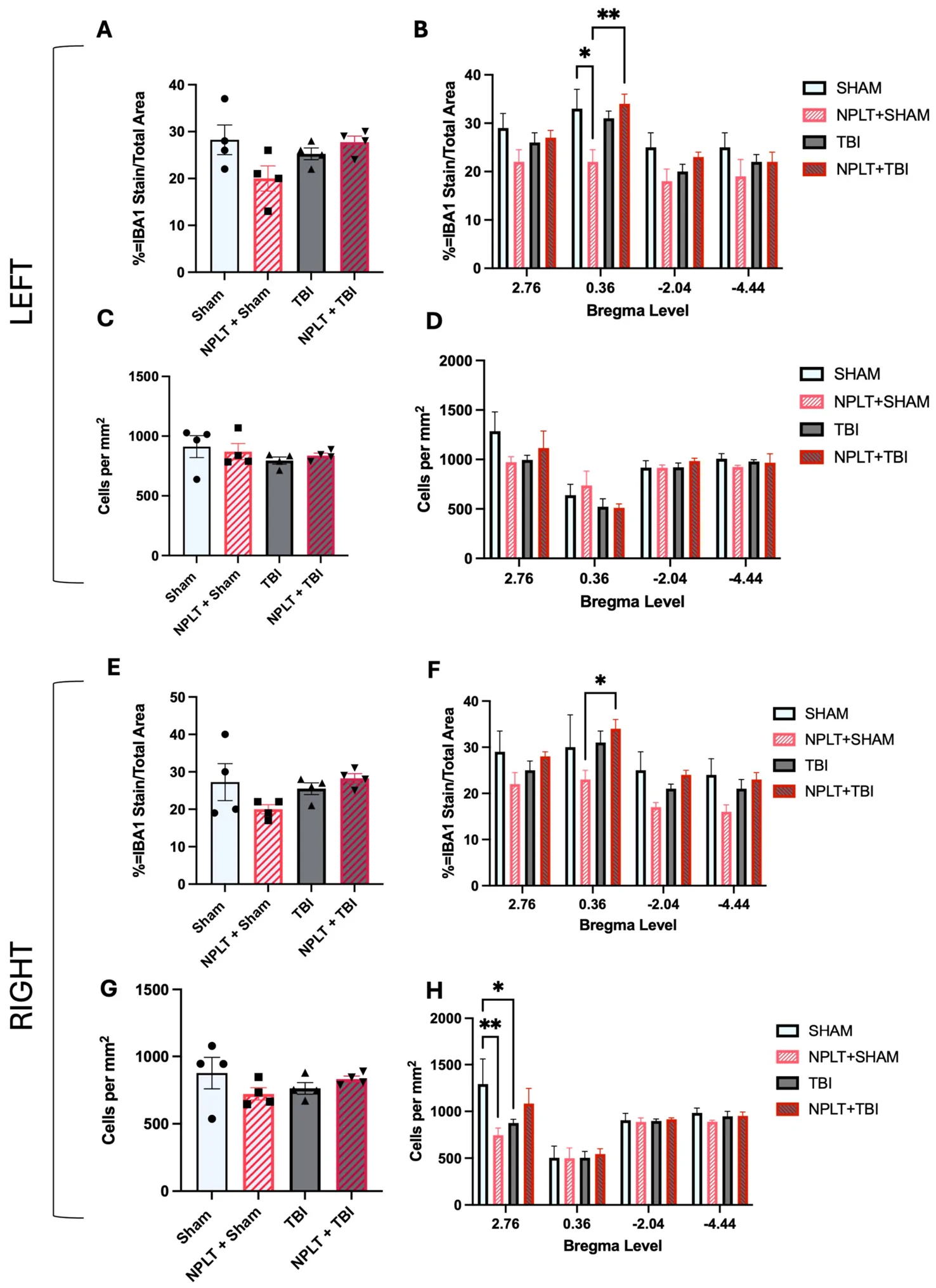
Microglia in the somatosensory cortex. Graphs (**A**–**D**) show Iba1 percent stain (**A**,**B**) and cell count (**C**,**D**) in the left hemisphere of the somatosensory cortex, while (**E**–**H**) show the same measures in the right hemisphere of the somatosensory cortex. Data is presented as mean +/− SEM. * *p* < 0.05, ** *p* < 0.01 one-way ANOVA with Tukey’s post hoc test.

**Figure 9:**
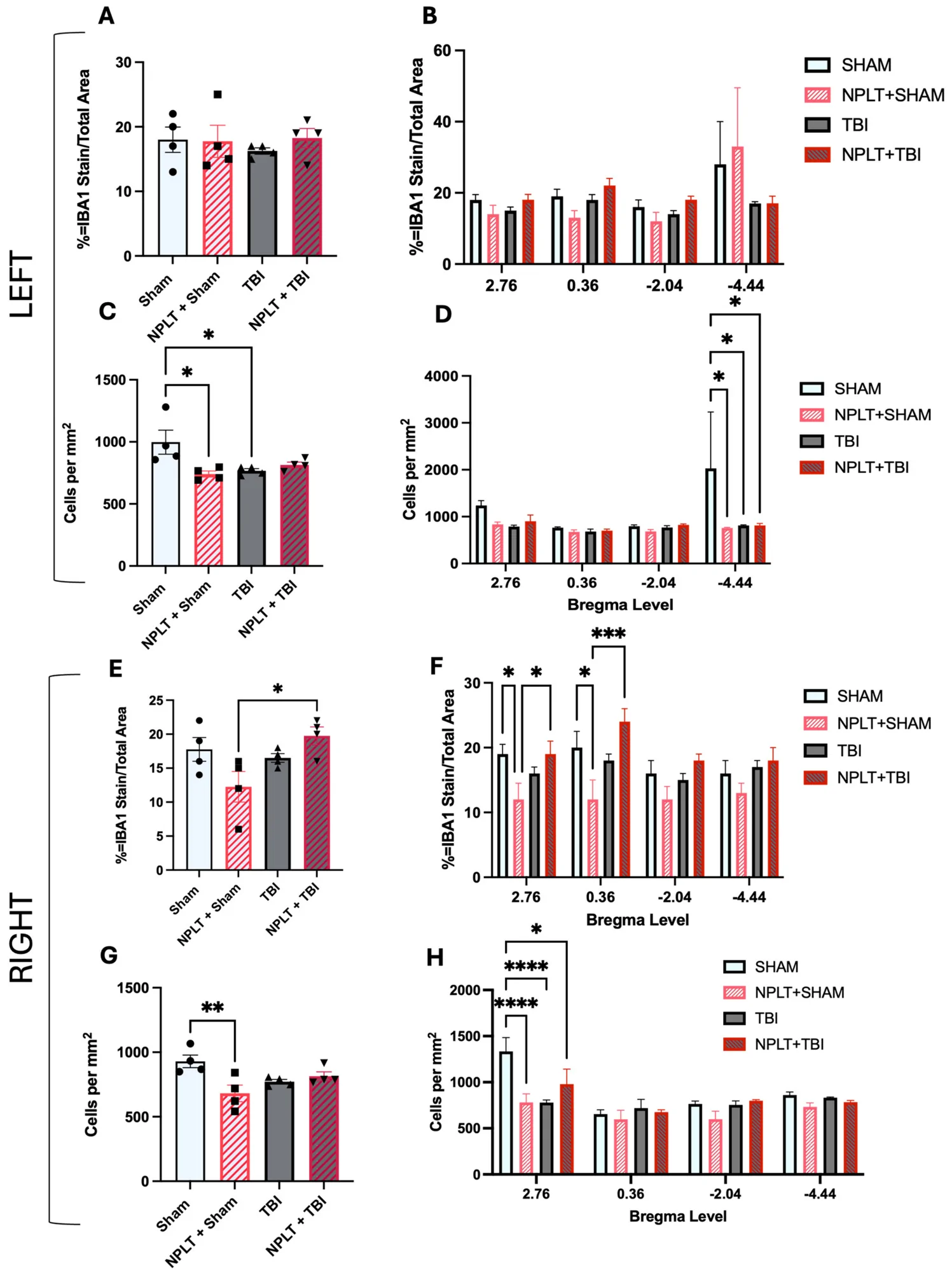
Microglia in the corpus callosum. Graphs (**A**–**D**) show Iba1 percent stain (**A**,**B**) and cell count (**C**,**D**) in the left hemisphere of the corpus callosum, while (**E**–**H**) show the same measures in the right hemisphere of the corpus callosum. Data is presented as mean +/− SEM. * *p* < 0.05, ** *p* < 0.01, *** *p* < 0.001, **** *p* < 0.0001 one-way ANOVA with Tukey’s post hoc test.

**Figure 10:**
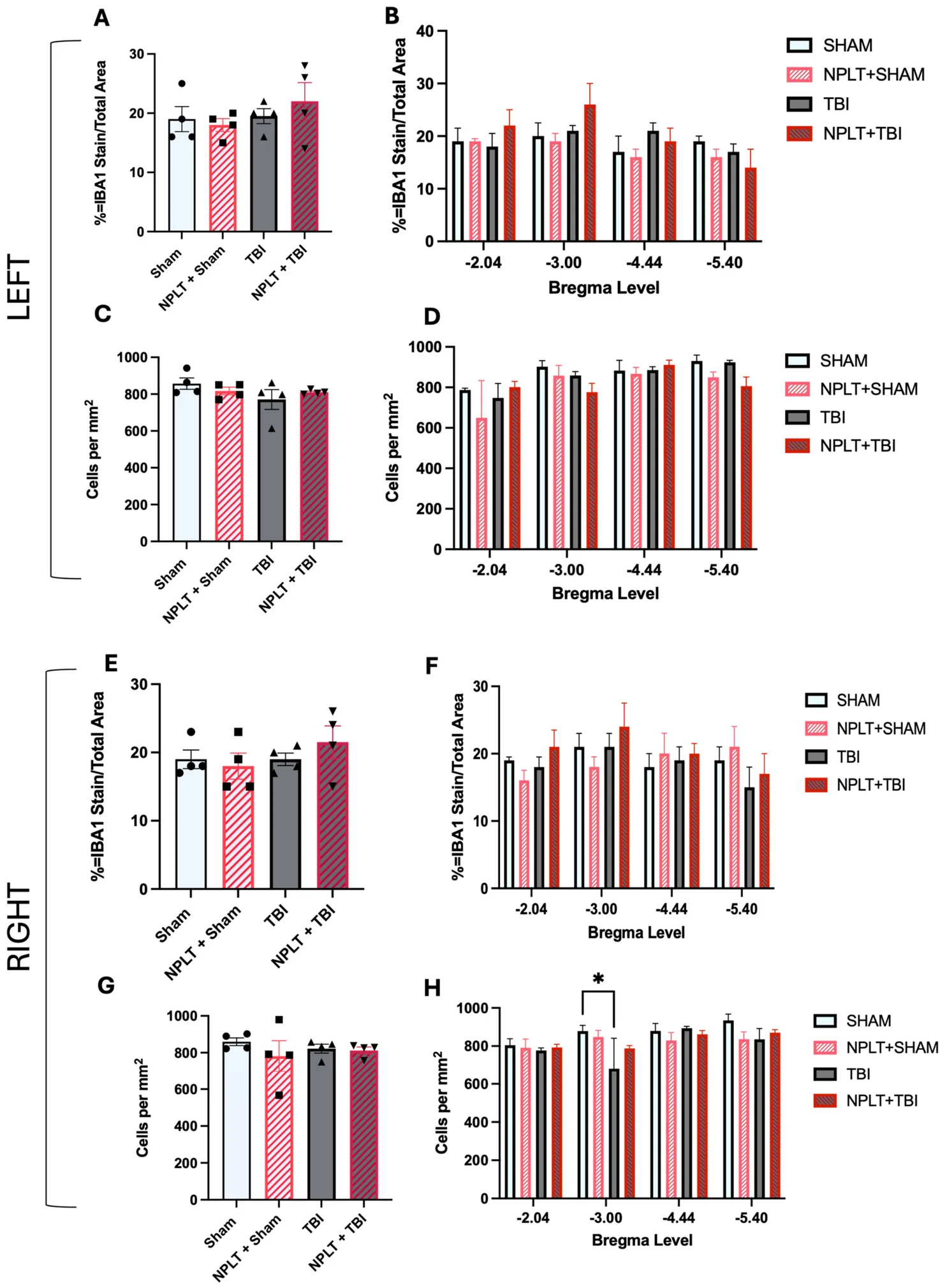
Microglia in the thalamus. Graphs (**A**–**D**) show Iba1 percent stain (**A**,**B**) and cell count (**C**,**D**) in the left hemisphere of the thalamus, while (**E**–**H**) show the same measures in the right hemisphere of the thalamus. Data is presented as mean +/− SEM. * *p* < 0.05 one-way ANOVA with Tukey’s post hoc test.

**Figure 11:**
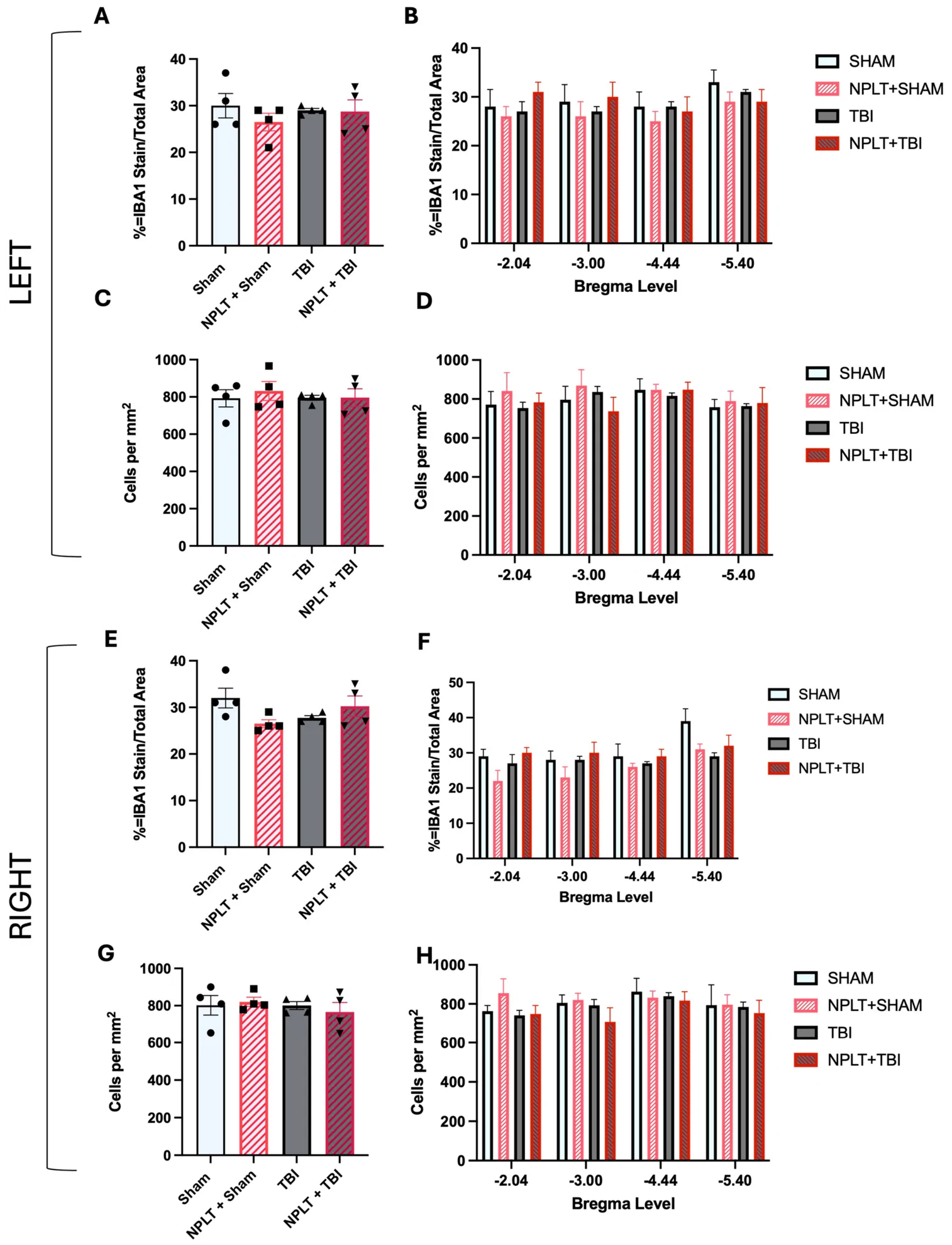
Microglia in the hippocampus. Graphs (**A**–**D**) show Iba1 percent stain (**A**,**B**) and cell count (**C**,**D**) in the left hemisphere of the hippocampus, while (**E**–**H**) show the same measures in the right hemisphere of the hippocampus. Data is presented as mean +/− SEM. One-way ANOVA with Tukey’s post hoc test.

To evaluate the effect of NPLT and TBI on inflammation, brain sections were immunostained using an antibody against Iba1 (a marker for microglia) and quantified by measuring the number of Iba1+ cells by area and percent-stained area in the motor cortex (Figure 7), somatosensory cortex (Figure 8), corpus callosum (Figure 9), thalamus (Figure 10), and hippocampus (Figure 11).

#### 2.5.1. Motor Cortex: NPLT Produces Distinct Region- and Treatment-Dependent Microglial Responses

In the left motor cortex, there were no significant differences observed among groups in overall percent Iba1+ stain per total area, percent stain by bregma level, and overall Iba1+ cell count per area. Cell count by bregma level was significant at bregma 2.76. NPLT + Sham and TBI were decreased compared to Sham (*p* = 0.0234 and *p* = 0.0139, respectively), although NPLT + TBI was not significantly different from Sham. Notably, in the left hemisphere for both metrics and percent stain by bregma level, there was a consistent trend of changes: NPLT + Sham was reduced compared to all groups and TBI was reduced compared to NPLT + TBI. This did not hold in cell count by bregma level, as NPLT + TBI was increased compared to TBI.

In the right motor cortex, NPLT + Sham was significantly reduced compared to Sham (*p* = 0.0374) in overall percent Iba1+ stain per total area, but no significant differences among groups were observed in overall Iba1+ cell count per area. By bregma level, at bregma 0.36, NPLT + Sham was significantly reduced in percent stain compared to Sham (*p* = 0.0321) and NPLT + TBI (*p* = 0.0131) and non-significantly compared to TBI. At bregma −2.04, NPLT + Sham was significantly reduced compared to Sham (*p* = 0.0321) and non-significantly reduced compared to TBI and NPLT + TBI. NPLT + Sham is similarly reduced in bregma 2.76 but non-significantly. Interestingly, these relationships mirrored the changes in percent stain taking place in the right hemisphere. Sham, TBI, and NPLT + TBI are equal. Cell count by bregma level was significantly reduced in NPLT + Sham and TBI compared to Sham (*p* = 0.0033 and *p* = 0.0045, respectively) at bregma 2.76. In both hemispheres for percent stain and left cell count, there was a consistent trend of changes: NPLT + Sham was reduced compared to all groups and TBI was reduced compared to NPLT + TBI. This did not hold in cell count in the right hemisphere, as TBI was further reduced compared to NPLT + Sham.

#### 2.5.2. Somatosensory Cortex: Microglial Responses Show Bregma-Dependent Modulation by NPLT

In the left somatosensory cortex, there were no significant differences in average Iba1+ percent stain per total area among groups. By bregma level, at bregma 0.36, Iba1+ percent stain was decreased in NPLT + Sham vs. Sham (*p* = 0.0135) and NPLT + TBI (*p* = 0.0060). It was non-significantly decreased compared to TBI. This relationship of changes held across the hemisphere and all bregma levels. NPLT + TBI was non-significantly increased to TBI in the hemisphere and across all bregma levels except for −4.44, where group means were equal. At bregma −2.04, Iba1+ percent stain was non-significantly decreased in NPLT + Sham vs. Sham. There were no differences in average Iba1+ cell count per area in the left somatosensory cortex nor across bregma levels.

In the right somatosensory cortex, no significant differences in average Iba1+ percent stain were observed. At bregma 0.36, Iba1+ percent stain was decreased in NPLT + Sham vs. NPLT + TBI (*p* = 0.0475). There were no significant differences in average Iba1+ cell count per area; however, at bregma 2.76, Iba1+ cell count was decreased in Sham vs. NPLT + Sham (*p* = 0.0016) and TBI (*p* = 0.0226). Notably, NPLT + TBI did not significantly differ from Sham. Interestingly, group changes trended similarly in the right hemisphere in both percent stain and cell count, holding across bregma levels: NPLT + Sham tended to be decreased compared to all groups, while TBI tended to be slightly increased compared to NPLT + Sham but not NPLT + TBI. This relationship trended across left and right hemispheres in percent stain, but not left hemispheric cell count, as NPLT + Sham was mildly increased compared to TBI and NPLT + TBI.

#### 2.5.3. Corpus Callosum: NPLT Differentially Modulates Microglial Responses Across Bregma Levels

In the left corpus callosum, there were no significant differences between treatment groups in overall percent Iba1+ stain per total area. There were significant reductions in overall Iba1+ cell count per area for NPLT + Sham and TBI compared to Sham (*p* = 0.0201 and *p* = 0.037, respectively). Notably, NPLT + TBI did not significantly differ from Sham. By bregma level, there were no significant differences in percent stain, but bregma −4.44 showed significant differences in cell count per area. NPLT + Sham, TBI, and NPLT + TBI cell counts were significantly reduced compared to Sham (*p* = 0.0254, *p* = 0.0347, and *p* = 0.0347, respectively).

In the right corpus callosum, overall percent Iba1+ stain per total area in NPLT + Sham was significantly decreased compared to NPLT + TBI (*p* = 0.0276). There was also a significant reduction in overall Iba1+ cell count per area in NPLT + Sham compared to Sham (*p* = 0.0096). By bregma level, percent stain was significantly decreased in NPLT + Sham compared to Sham and NPLT + TBI (*p* = 0.0490 and *p* = 0.0490, respectively) at bregma 2.76 and bregma 0.36 (*p* = 0.0188 and *p* = 0.0002, respectively). This trend continued at bregma −2.04 and −4.44 but non-significantly. Iba1+ cell count per area by bregma level decreased in NPLT + Sham compared to Sham across all bregma levels, although these differences were only significant at bregma 2.76 (*p* < 0.0001). At this level, TBI and NPLT + TBI were also significantly decreased compared to Sham (*p* < 0.0001 and *p* = 0.0104, respectively).

#### 2.5.4. Thalamus: Microglial Responses Are Largely Unchanged Across Groups

In the left thalamus, no significant differences across treatment groups were observed in average Iba1+ percent stain per total area and average Iba1+ cell count per area. Furthermore, there were no differences observed across bregma levels. In the right thalamus, the only significant difference was seen at bregma −3.00, such that Iba1+ cell count was decreased in TBI vs. Sham (*p* = 0.0417). Of note, similar trends were seen in Iba1+ percent stain per total area in the left vs. the right hemisphere. Changes were mild, but NPLT + Sham decreased compared to Sham, while TBI and NPLT + TBI were increased. The same trend in percent stain appeared in bregma −3.00.

#### 2.5.5. Hippocampus: Minimal Microglial Changes Observed with Limited NPLT Effect

There were no significant differences across treatment groups in average Iba1+ percent stain per total area and average Iba1+ cell count per area in either hemisphere or across bregma levels. Although changes were again mild, similar trends were seen across hemispheres. Percent stain per area decreased the most in NPLT + Sham, followed by TBI and NPLT + TBI compared to Sham. Opposingly, average cell count per area increased in NPLT + Sham followed by TBI and NPLT + TBI.

### 2.6. Prophylactic NPLT Differentially Modulates Region-Dependent Astrocytic Responses Following Mild Blast Injury

**Figure 12:**
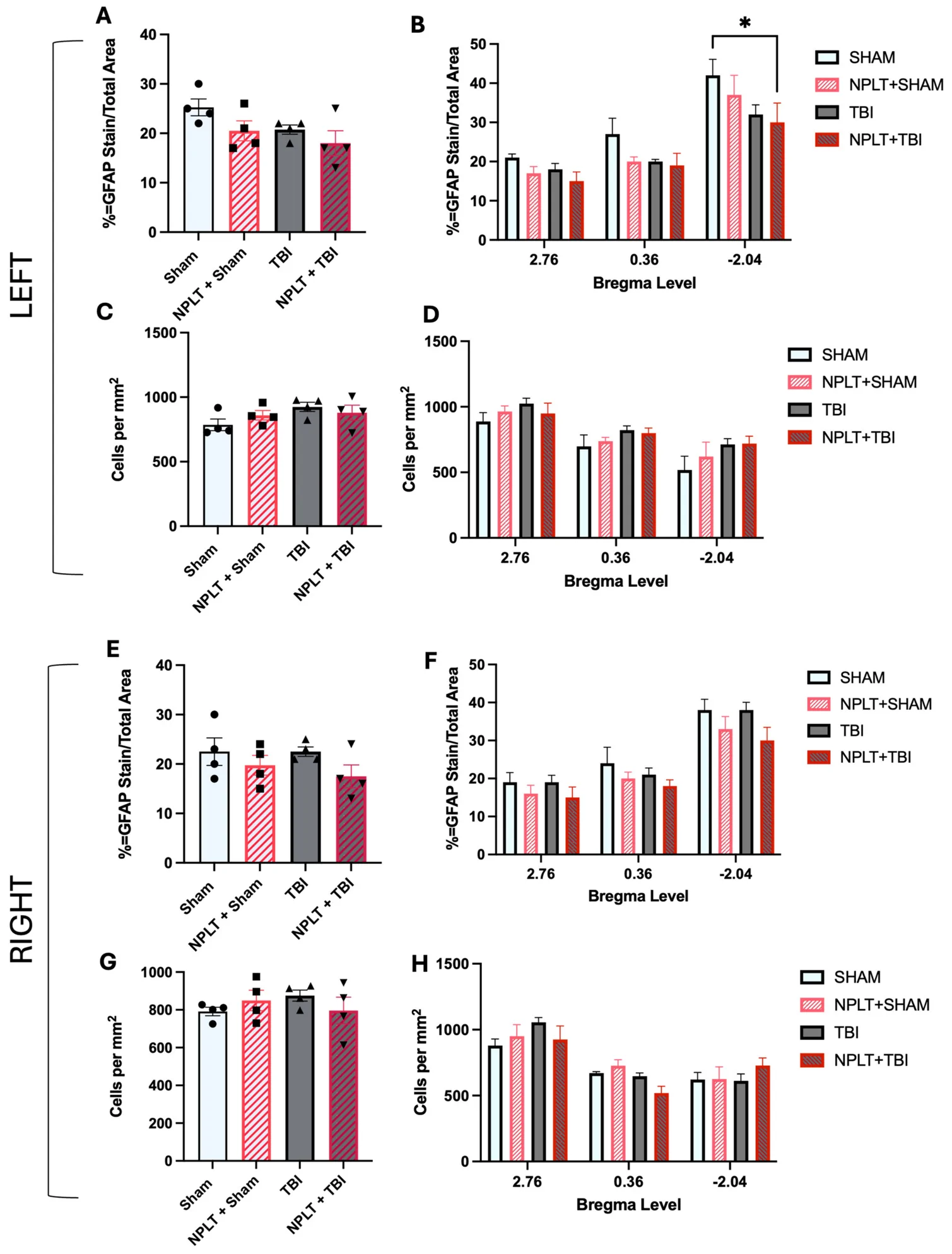
Astrocytes in the motor cortex. Graphs (**A**–**D**) show GFAP percent stain (**A**,**B**) and cell count (**C**,**D**) in the left hemisphere of the motor cortex, while (**E**–**H**) show the same measures in the right hemisphere of the motor cortex. Data is presented as mean +/− SEM. * *p* < 0.05 one-way ANOVA with Tukey’s post hoc test.

**Figure 13:**
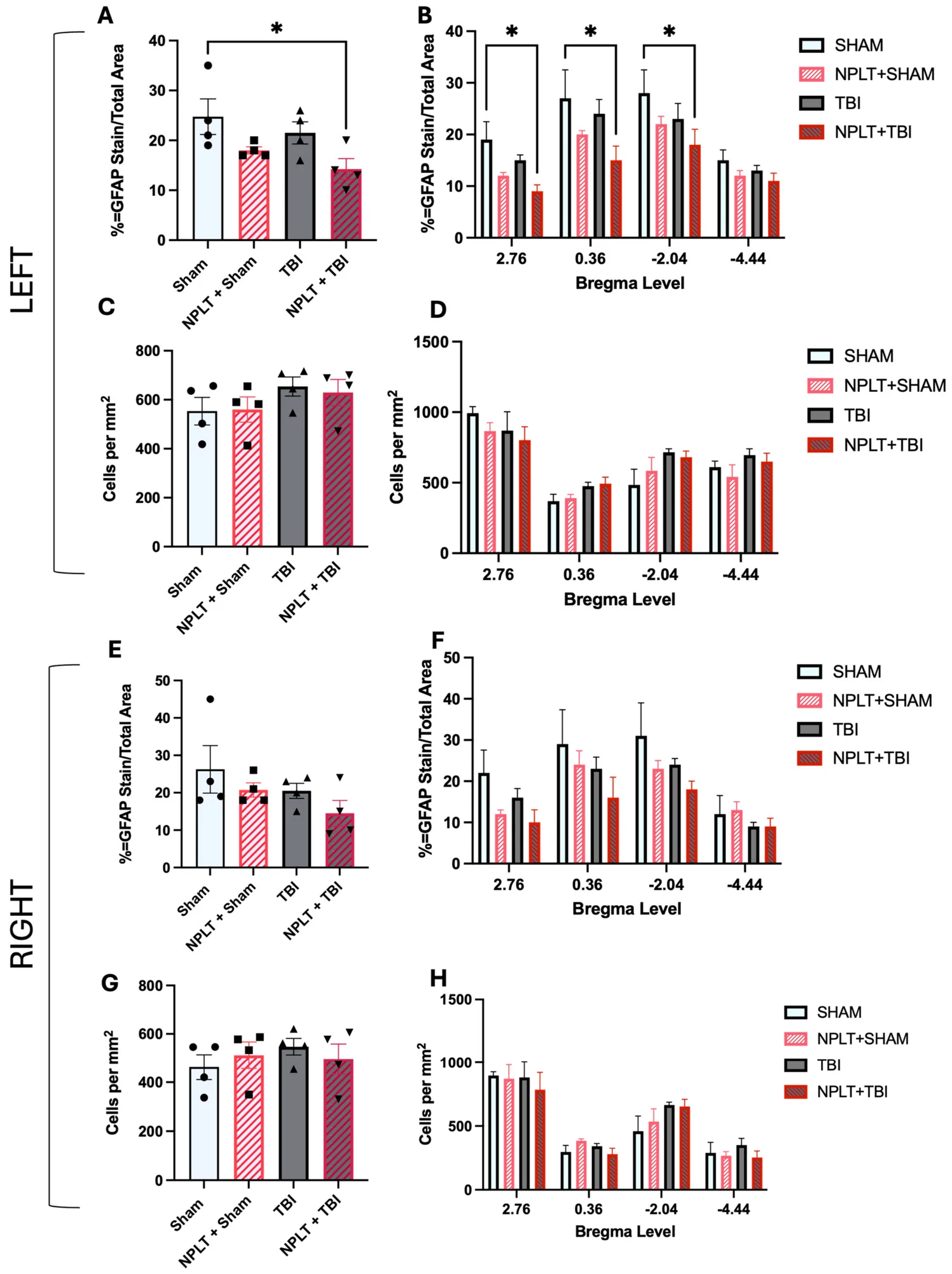
Astrocytes in the somatosensory cortex. Graphs (**A**–**D**) show GFAP percent stain (**A**,**B**) and cell count (**C**,**D**) in the left hemisphere of the somatosensory cortex, while (**E**–**H**) show the same measures in the right hemisphere of the somatosensory cortex. Data is presented as mean +/− SEM. * *p* < 0.05 one-way ANOVA with Tukey’s post hoc test.

**Figure 14:**
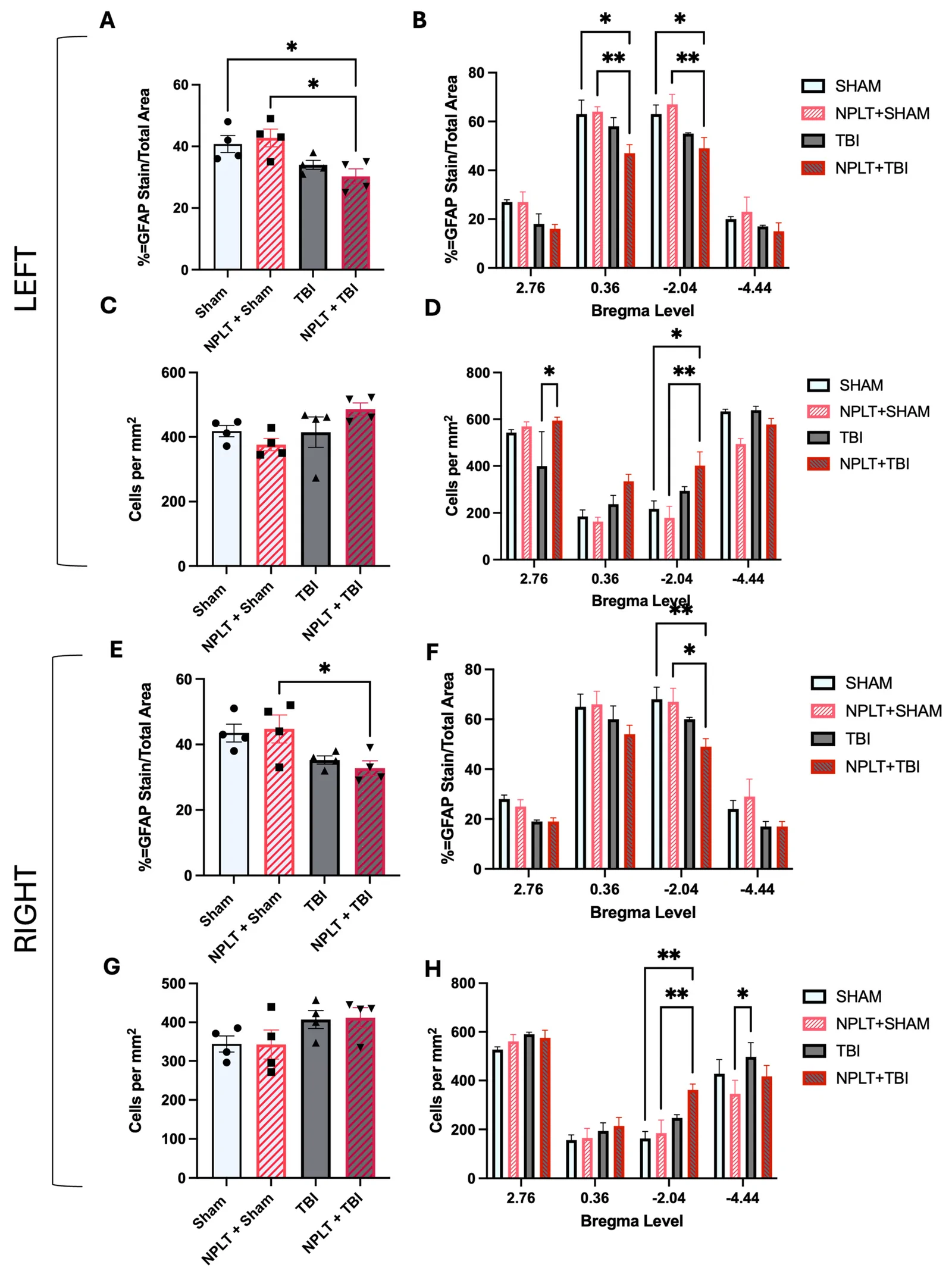
Astrocytes in the corpus callosum. Graphs (**A**–**D**) show GFAP percent stain (**A**,**B**) and cell count (**C**,**D**) in the left hemisphere of the corpus callosum, while (**E**–**H**) show the same measures in the right hemisphere of the corpus callosum. Data is presented as mean +/− SEM. * *p* < 0.05, ** *p* < 0.01 one-way ANOVA with Tukey’s post hoc test.

**Figure 15:**
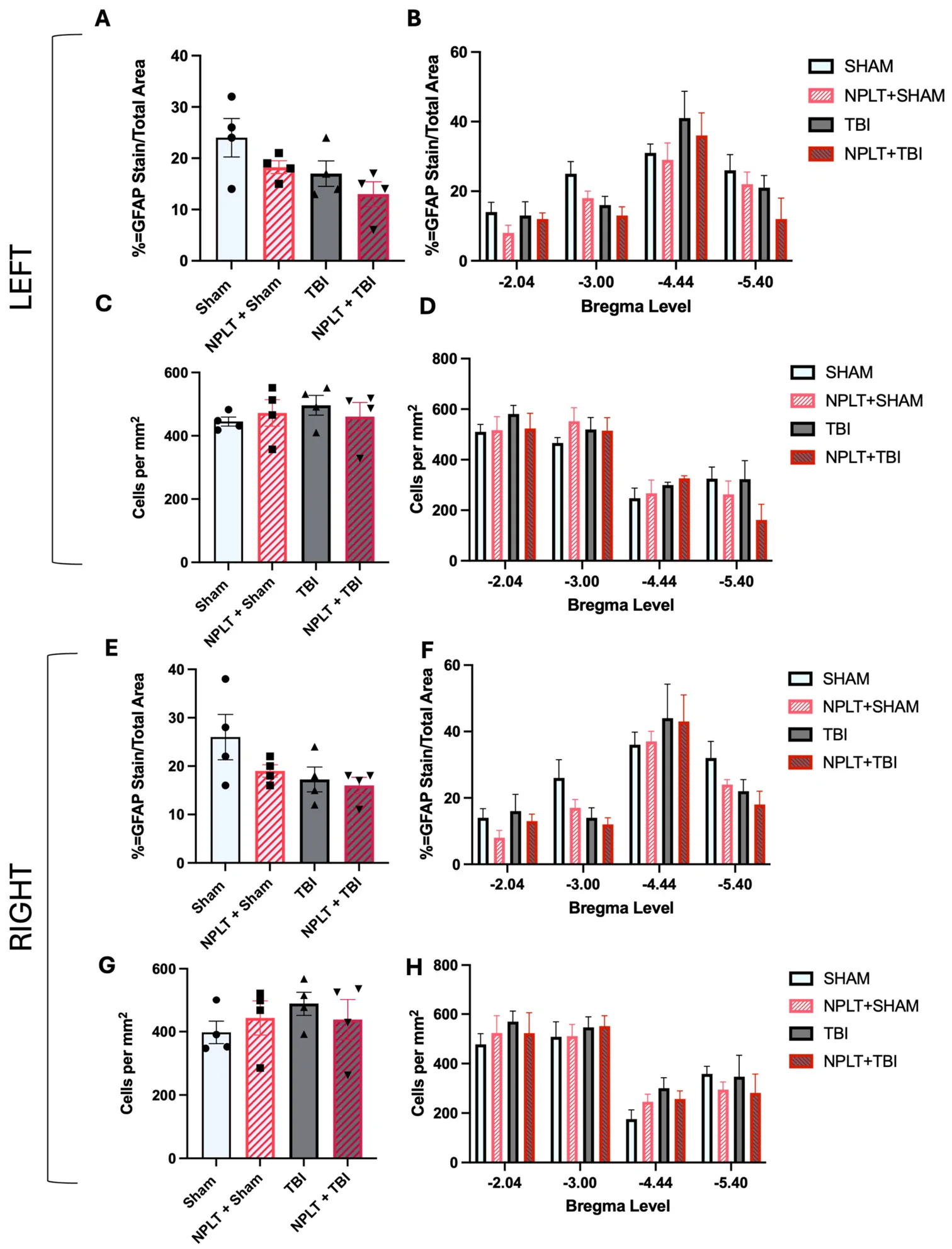
Astrocytes in the thalamus. Graphs (**A**–**D**) show GFAP percent stain (**A**,**B**) and cell count (**C**,**D**) in the left hemisphere of the thalamus, while (**E**–**H**) show the same measures in the right hemisphere of the thalamus.

**Figure 16:**
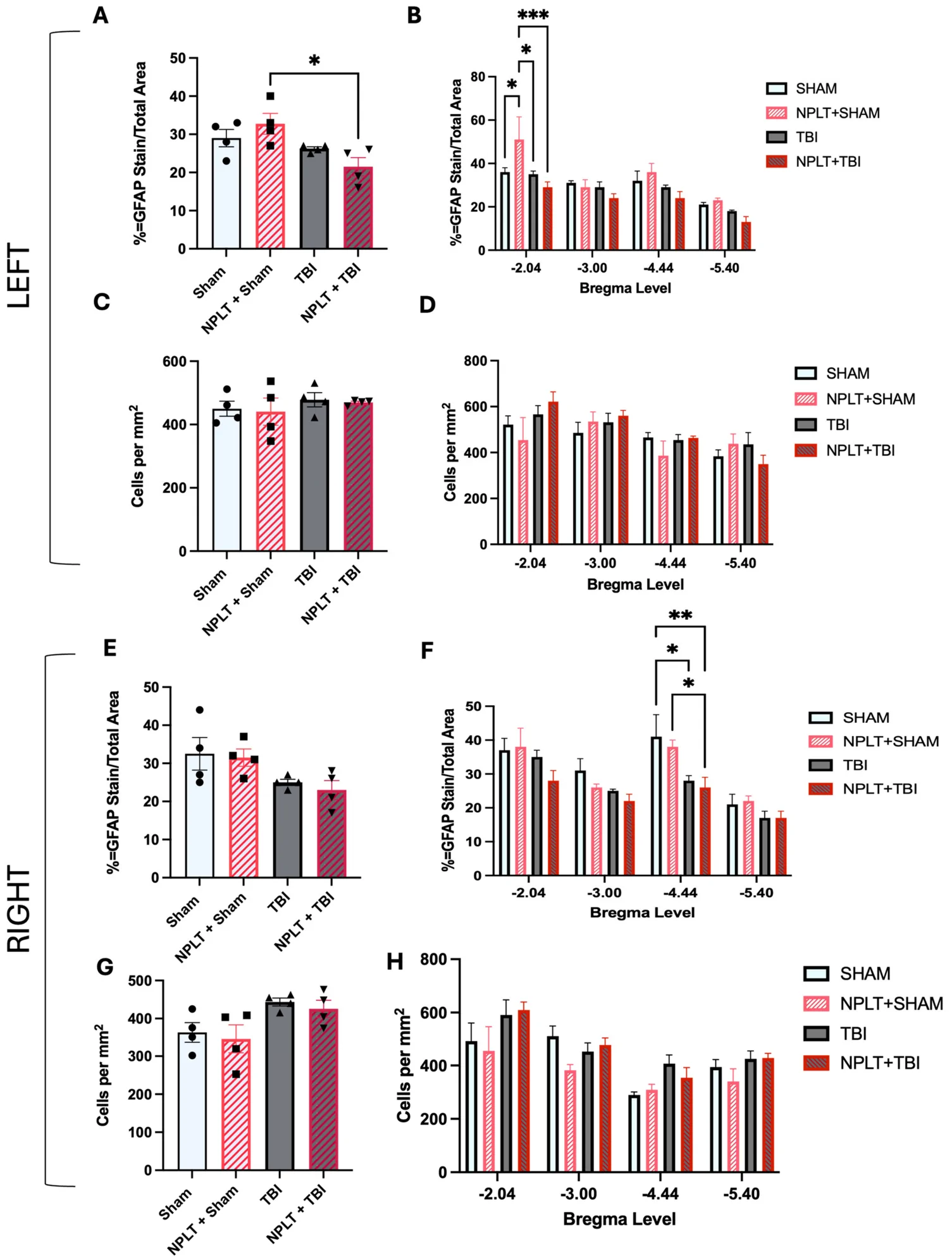
Astrocytes in the hippocampus. Graphs (**A**–**D**) show GFAP percent stain (**A**,**B**) and cell count (**C**,**D**) in the left hemisphere of the hippocampus, while (**E**–**H**) show the same measures in the right hemisphere of the hippocampus. Data is presented as mean +/− SEM. * *p* < 0.05, ** *p* < 0.01, *** *p* < 0.001 one-way ANOVA with Tukey’s post hoc test.

To evaluate for astrocytosis, brain tissue was stained with GFAP in the motor cortex (Figure 12), somatosensory cortex (Figure 13), corpus callosum (Figure 14), thalamus (Figure 15), and hippocampus (Figure 16).

#### 2.6.1. Motor Cortex: Astrocytic Responses Differ by Treatment and Quantitative Measure

In the left motor cortex, there were no significant differences in overall percent GFAP+ stain per total area. There was a non-significant trend for a mild decrease in TBI followed by NPLT + Sham and NPLT + TBI compared to Sham. Overall GFAP+ cell count per area increased in TBI compared to Sham, while NPLT + Sham and NPLT + TBI were mildly decreased compared to TBI. These trends were also observed across various bregma levels. Percent stain per total area at bregma −2.04 was significantly decreased in NPLT + TBI compared to Sham (*p* = 0.0409). GFAP+ cell count per area across bregma levels was non-significant; however, the trends were consistent across all three levels and the whole hemisphere. In the right motor cortex, there were no significant differences observed in the hemispheric percent GFAP+ stain per total area and GFAP+ cell count per area nor by bregma level in either metric. Notably, the hemispheric trends mirror those seen in the left hemisphere.

#### 2.6.2. Somatosensory Cortex: NPLT + TBI Is Associated with Reduced GFAP Staining Following Mild Blast

In the left somatosensory cortex, average GFAP+ percent stain per total area was decreased in NPLT + TBI vs. Sham (*p* = 0.0380). By bregma level, at bregma 2.76, 0.36, and −2.04, GFAP+ percent stain was significantly decreased in NPLT + TBI vs. Sham (*p* = 0.0462, *p* = 0.0115, and *p* = 0.0462, respectively) and non-significantly in NPLT + Sham and TBI. At bregma −4.44, the same relationship of changes occurred, albeit non-significantly. No significant differences in average hemispheric GFAP+ cell count per area were observed, nor by bregma level; however, similar relationships of change occurred, particularly in the whole hemisphere and at bregma levels 0.36, −2.04, and −4.44.

In the right somatosensory cortex, no significant differences in average GFAP+ percent stain, by hemisphere and bregma level, were observed, although the group changes tended to mirror what was observed in the left hemisphere. There were no significant differences in average GFAP+ cell count per area and across bregma levels; however, there were similar trends of change in the whole hemisphere and at bregma levels 2.76, −2.04, and −4.44 compared to the left hemisphere.

#### 2.6.3. Corpus Callosum: NPLT Differentially Alters GFAP Staining and Astrocyte Density

In the left corpus callosum, overall percent GFAP+ stain per total area was non-significantly decreased in TBI and significantly decreased in NPLT + TBI compared to Sham (*p* = 0.0467) and NPLT + Sham (*p* = 0.0171). Overall GFAP+ cell count per area was decreased in NPLT + Sham compared to Sham, followed by an increase in TBI and NPLT + TBI. By bregma level, percent stain was significantly decreased in NPLT + TBI compared to Sham and NPLT + Sham (*p* = 0.0132 and *p* = 0.0076, respectively) and non-significantly to TBI at bregma 0.36. The same relationship occurred at bregma −2.04: NPLT + TBI was decreased compared to Sham and NPLT + Sham (*p* = 0.00371 and *p* = 0.0043) and non-significantly to TBI. By bregma level, GFAP+ cell count at bregma 2.76 was decreased in TBI compared to NPLT + TBI (*p* = 0.0244), but NPLT + TBI did not significantly differ from Sham and NPLT + Sham. NPLT + TBI was increased compared to Sham and NPLT + Sham (*p* = 0.0365 and *p* = 0.0075) at bregma −2.04.

In the right corpus callosum, overall percent GFAP+ stain per total area was non-significantly decreased in TBI and significantly decreased in NPLT + TBI compared to NPLT + Sham (*p* = 0.0484) and non-significantly to Sham, mirroring the same trend seen in the left hemisphere. There were no significant differences between treatment groups in the overall GFAP+ cell count per area but the relationship of changes mirrored those seen on the left side. By bregma level, similar changes were seen compared to the left: at bregma −2.04, percent stain was significantly decreased in NPLT + TBI compared to Sham and NPLT + Sham (*p* = 0.0062 and *p* = 0.0103, respectively). At bregma 0.36, there were no significant differences, but the relationship of changes among groups was the same between left and right hemispheres, as it was at bregma −4.44. At bregma −2.04, GFAP+ cell count was increased in NPLT + TBI compared to Sham (*p* = 0.0027), NPLT + Sham (*p* = 0.0086), and non-significantly to TBI. At bregma −4.44, cell count was increased in TBI compared to NPLT + Sham (*p* = 0.0307), mirroring the same non-significant trend on the left side.

#### 2.6.4. Thalamus: Astrocytic Responses Show Limited Modulation by NPLT

In the left thalamus, average GFAP+ percent stain per total area was non-significantly decreased in all groups compared to Sham. The largest decrease occurred in NPLT + TBI, followed by TBI and NPLT + Sham. Significant differences were not observed in average GFAP+ cell count per area, although there was a non-significant increase in TBI accompanied by a decrease in NPLT + TBI and NPLT + Sham. There were no bregma-level significant differences seen in GFAP+ percent stain and average GFAP+ cell count per area. In the right thalamus, the same trend emerged: average GFAP+ percent stain per total area was decreased in all groups compared to Sham, with the largest decrease in NPLT + TBI, followed by TBI and NPLT + Sham, respectively. Interestingly, the trend in cell count per area on the right followed that of the left: there was a non-significant increase in TBI accompanied by a decrease in NPLT + TBI and NPLT + Sham. There were no significant differences by bregma level on the left side in either metric.

#### 2.6.5. Hippocampus: NPLT and Blast Produce Distinct Astrocytic Responses

In the left hippocampus, average GFAP+ percent stain per total area was observed to increase in NPLT + Sham followed by a decrease in TBI and NPLT + TBI compared to Sham. The decrease in NPLT + TBI vs. NPLT + Sham was statistically significant (*p* = 0.0141). By bregma level, at bregma −2.04, GFAP+ percent stain was increased in NPLT + Sham vs. Sham (*p* = 0.0219), TBI (*p* = 0.0129), and NPLT + TBI (*p* = 0.0004). No significant differences in average GFAP+ cell count per area, including across bregma levels, were observed.

In the right hippocampus, average GFAP+ percent stain per total area followed the same trend non-significantly: there was a mild change in NPLT + Sham followed by a decrease in TBI and NPLT + TBI compared to Sham. By bregma level, at bregma −4.44, GFAP+ cell count was decreased in TBI vs. Sham (*p* = 0.0212) and NPLT + TBI vs. Sham (*p* = 0.0060) and NPLT + Sham (*p* = 0.0381). No significant differences in average GFAP+ cell count per area were observed, nor across bregma levels; however, similar trends in average cell count appeared in both hemispheres: there was a mild decrease in NPLT + Sham compared to Sham followed by an increase in TBI and NPLT + TBI.

### 2.7. Prophylactic NPLT Does Not Prevent Localized Blast-Associated Reductions in Corpus Callosum Myelin Staining

**Figure 17:**
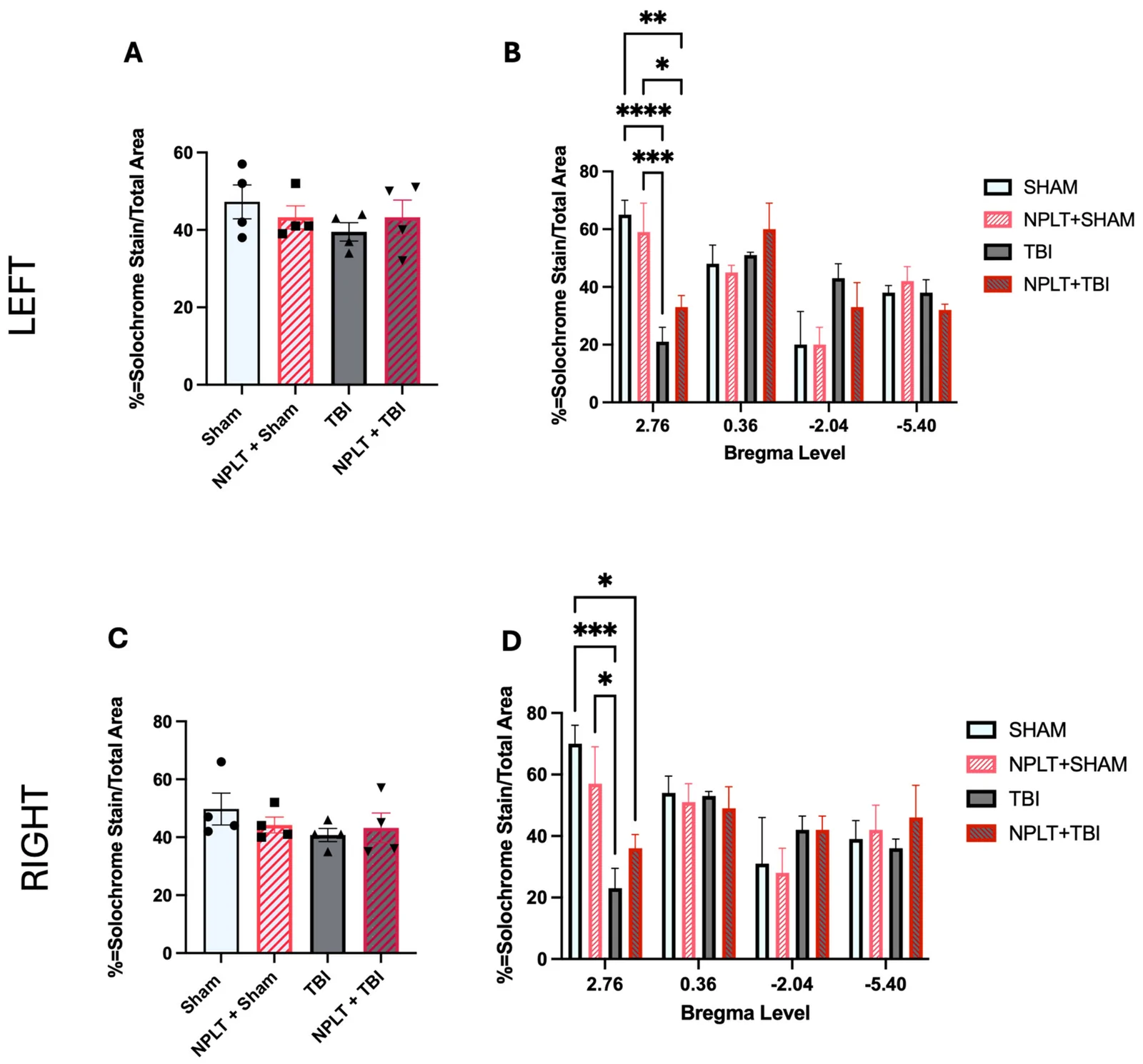
Myelination staining in the corpus callosum. Graphs (**A**,**B**) show Solochrome percent stain in the left hemisphere of the corpus callosum, while (**C**,**D**) show Solochrome percent stain in the right hemisphere of the corpus callosum. Data is presented as mean +/− SEM. * *p* < 0.05, ** *p* < 0.01, *** *p* < 0.001, **** *p* < 0.0001 one-way ANOVA with Tukey’s post hoc test.

To assess damage to myelination post-bTBI, Solochrome was measured in the right and left corpus callosum as percent stain per total area (Figure 17). There were no significant differences between the treatment groups in overall average percent stain in both the left and the right corpus callosum. Reviewing the location by bregma level (2.76, 0.36, −2.04, and −5.40), there was a significant decrease in percent stain per total area in TBI and NPLT + TBI compared to Sham (*p* < 0.0001 and *p* = 0.0036, respectively) and NPLT + Sham (*p* = 0.0004 and *p* = 0.0239, respectively) at bregma 2.76 in the left corpus callosum. There was also a significant decrease in NPLT + TBI compared to Sham at the same bregma level (*p* = 0.0007). On the right side, at bregma 2.76, TBI and NPLT + TBI percent stain per total area were decreased compared to Sham (*p* = 0.0003 and *p* = 0.0129, respectively). TBI was significantly decreased compared to NPLT + Sham (*p* = 0.0129). There were no significant differences at bregma −2.04. Notably, similar trends were seen on the left and right sides, such that there was a non-significant mild decrease in TBI accompanied by mild increases compared to Sham in NPLT + TBI and NPLT + Sham.

## 3. Discussion

Blast TBI is a unique injury characterized by exposure to a propagating blast wave, the outcome of which can be classified as mild, moderate, or severe. In humans, and disproportionately military service members, mild blast injury can manifest as mild or transient cognitive, emotional, vestibulomotor, and neurological impairment [17], but the transient nature of symptoms does not preclude an individual from long-term suffering, as 30–50% report symptoms one year post-injury [18]. On a cellular level, blast TBI results in an array of pathological changes that contribute to a complex disease process, including axonal damage and neuroinflammation [19]. In this study, we report on the efficacy of a single application of a novel, patented, non-invasive therapy that combines NIL (808 nm) and US to generate optoacoustic pulses within the brain as a prophylactic treatment twenty-four hours before blast-induced TBI in Sprague-Dawley rats. Overall, prophylactic (PrEP) NPLT was associated with benefit to cognitive performance, modest effects on fine motor function, and minimal effect on gross neurological function. Histological responses were heterogenous and depended on region, bregma level, and quantitative measure.

### 3.1. Cognitive Preservation and Attenuation of Motor Dysfunction Despite Persistent Histopathology

We first evaluated neurological and motor function, including reflexes, gross motor function, and fine vestibular coordination, via Neuroscore, beam walk, and beam balance, respectively. A single mild blast did not produce significant between-group differences in Neuroscore, or beam balance, consistent with the relatively subtle neurological phenotype expected following mild injury. The beam walk was more sensitive to blast-associated dysfunction. TBI animals tended to perform more poorly than Sham or treated animals across four of five post-injury testing days and performed significantly worse on PIDs 1, 3, 4, and 5 compared with their pre-injury baseline. PrEP NPLT appeared to attenuate this dysfunction, as NPLT + TBI animals generally outperformed TBI animals, although the between-group difference reached significance only on PID 4. Thus, the motor findings suggest a modest benefit of PrEP NPLT rather than complete prevention of neurological dysfunction. One possible reason for the modest magnitude of these two trends is the therapeutic protocol of NPLT. Rats were treated once, 24 h prior to injury. It is possible that additional PrEP treatments are needed prior to injury to maximize efficacy; however, additional study is needed to fully elucidate the most effective therapeutic protocol.

Next, we evaluated NPLT’s ability to ameliorate blast-induced cognitive dysfunction, namely, working memory and spatial learning via the MWM. Working memory, i.e., T2 latencies, was significantly impaired in TBI compared to Sham on the first day of testing, PID 13. Notably, this significant difference was absent between Sham and NPLT + TBI. TBI latency remained elevated relative to Sham throughout testing and again differed significantly on PID 17. NPLT + TBI performed incrementally better each day, although did not outperform TBI again until the last day of testing. While all groups outperformed their first-day performance on the second day of testing, a typical finding attributed to the successful learning of the task [20], all groups except TBI showed significantly increased performance on the last day of testing compared to their second day of testing. Based on these findings, cognitive performance was most consistently associated with benefit from PrEP NPLT in this study. That said, subtle beam-walk impairment was also observed after blast, so differences in MWM latency cannot be assumed to reflect cognition exclusively.

Swimming speed was not quantified in the present study; therefore, although the MWM findings are consistent with preservation of cognitive performance, a contribution of treatment- or injury-related differences in swimming ability to platform latency cannot be excluded.

An important question raised by these findings is how a single five-minute NPLT treatment administered twenty-four hours prior to injury could influence cognitive behavior despite only modest structural changes. Findings from Study 1 provide initial evidence for underlying molecular changes that occur after NPLT treatment and are sustained for at least 24 h post-treatment. Hippocampal BDNF mRNA was significantly increased in uninjured rats treated with NPLT compared with Sham-treated animals. BDNF is a key mediator of activity-dependent synaptic plasticity in the hippocampus and plays an important role in hippocampus-dependent learning and memory. Experimental disruption of BDNF or its TrkB receptor impairs hippocampal long-term potentiation and memory formation, whereas BDNF signaling contributes to the maintenance of long-lasting synaptic plasticity [21,22,23]. Thus, the sustained increase in hippocampal BDNF observed after NPLT is consistent with the establishment of a molecular environment that may favor synaptic plasticity and functional resilience at the time of subsequent blast exposure. Although Study 1 does not establish BDNF as the causal mediator of behavioral protection following NPLT, it provides evidence that a single NPLT treatment, administered at the same interval used for PrEP NPLT, can induce sustained molecular changes in the hippocampus that may contribute to beneficial cellular and functional responses.

This in vivo finding supports the hypothesis that NPLT acts, in part, through cellular priming, whereby initial treatment induces adaptive changes that alter or blunt the response to a later injury. This interpretation is consistent with previous in vitro findings from our laboratory. Johnson et al. (2026; [24]) treated adult hippocampal neural stem cells (NSCs) with NPLT prior to differentiation and found that neurons subsequently generated from treated NSCs demonstrated significantly reduced amyloid-β oligomer binding and were resistant to amyloid-β-induced mitochondrial dysfunction. Furthermore, the study evidenced transcriptional changes in NSCs 24 h after treatment, and a subset of upregulated genes remained enriched up to five days afterward. These genes included those related to protein synthesis and processing, proteostasis, FoxO signaling, and metabolic and cell survival pathways [24].

Importantly, these findings should not be taken as a mechanistic explanation for this study, as NPLT’s potential as an adaptive primer is likely multifactorial. Of note, Johnson et al. did not find changes in BDNF pathways in vitro [24]. It is more likely that different cell populations engage multiple different pathways in response to NPLT. Direct assessment of genetic and molecular changes following PrEP NPLT and blast injury is necessary to determine whether changes seen by Johnson et al. also play a role in mediating the cognitive changes seen in this study.

A notable finding of the present study was a disconnect between cognitive performance and regional histopathology. Despite preservation of working-memory performance in NPLT + TBI animals, NPLT did not uniformly normalize blast-associated glial responses or localized alterations in myelin staining. Moreover, hippocampal microglial changes were surprisingly limited. No significant group differences in average hippocampal Iba1 percent staining or cell density were observed in either hemisphere or across bregma levels, although mild, directionally similar group patterns were present bilaterally. Thus, preservation of MWM performance cannot readily be attributed to suppression of overt hippocampal microglial pathology.

One possibility for the behavioral–histological disconnect is the preconditioning effect of NPLT discussed above. Hypothetically, a preconditioned neural environment could alter the subsequent response to blast and allow neural circuits to maintain function despite persistent structural and glial abnormalities. BDNF-mediated plasticity, maintenance of synaptic function, altered cellular metabolism, and enhanced stress-response capacity are potential contributors to such functional resilience, although these possibilities were not directly tested in the present study. In addition, blast injury can produce neurophysiological abnormalities that are not necessarily reflected by conventional histological measures. Mild blast exposure in rats has been shown to alter hippocampal synaptic excitability and short-term plasticity and has also been associated with altered resting-state functional connectivity across distributed neural networks [25]. Because spatial learning and working memory depend on coordinated activity across multiple brain regions, behavioral performance may not correlate directly with the magnitude of Iba1, GFAP, or myelin staining in any single region. In a recent meta-analysis, transcranial photobiomodulation (tPBM) was found to alter neural oscillations in brain waves, which was associated with a benefit to cognition, providing a potential explanation for preserved functional connectivity [26]. tPBM was found to both increase information processing speed and efficiency and to improve oscillation patterns shown to be present in various neurodegenerative diseases [27,28]. Consequently, PrEP NPLT could potentially preserve or facilitate compensatory neural function without completely preventing structural injury. Electrophysiological, synaptic, and functional connectivity studies will be needed to test this hypothesis.

### 3.2. Microglial Responses

Microglial changes to blast and NPLT were regionally heterogeneous. The most consistent changes were observed within cortical regions and the corpus callosum; alternatively, there was comparatively limited response in the thalamus and hippocampus. Interestingly, recurring non-significant trends were seen among treatment groups throughout hemispheres, bregma levels, and metrics. For example, NPLT + Sham animals frequently exhibited reduced Iba1 staining and/or cell density relative to Sham across cortical and callosal regions, while TBI and NPLT + TBI demonstrated distinct patterns depending on anatomical location. In the motor cortex, these directional relationships occurred across both hemispheres and multiple bregma levels, even where individual comparisons did not reach statistical significance. Similar recurring relationships were observed within the somatosensory cortex. These findings suggest that NPLT itself alters the microglial environment prior to injury, adding weight to the preconditioning hypothesis, and that subsequent blast exposure interacts with this altered state in a region-dependent manner.

Changes in Iba1+ cell density and percent staining did not always occur in parallel, suggesting that blast-associated microglial responses cannot be characterized solely by increases or decreases in microglial number. Cell density and percent staining are influenced by microglial activation state, and activated microglia can undergo changes in cellular morphology that alter the amount of tissue staining independent of cell count [29,30]. Thus, the discordance between Iba1+ cell density and percent staining observed here may reflect differences in the state of the resident microglial population. Microglial changes after TBI are complex and include changes in cell number, morphology, gene expression, and inflammatory phenotype [29,30]. Accordingly, present measures alone cannot establish the activation state or functional phenotype of the affected microglia. Overall, our findings demonstrate region-specific alterations in microglial density and Iba1 expression following blast and NPLT, but additional phenotype-specific and molecular analyses will be necessary to determine the functional significance of these responses.

PrEP NPLT appears to modulate microglial responses to blast injury rather than globally suppress or reverse. In several locations, NPLT + TBI measurements shifted relative to the change in the TBI, while in other locations it was recovered to not significantly differed from the Sham group. NPLT alone produced recurring changes in microglia, indicating PrEP treatment establishes a distinct baseline cellular change before injury rather than simply reversing post-injury inflammatory responses. This interpretation is compatible with the preconditioning hypothesis proposed above. Nevertheless, the current data do not establish microglial modulation as the mechanism responsible for cognitive preservation, particularly given the limited hippocampal microglial changes and persistence of other histopathological abnormalities. The exact mechanism of these changes is unknown. Previous studies using ultrasound (US) and near-infrared light (NIL) separately, although not applied prophylactically, indicate changes in microglial cell count occur after treatment [31,32], so it is not out of the question to think that the combination of these modalities would result in similar changes. Future research into the genomic and proteomic changes after NPLT treatment is needed to fully answer this open question.

### 3.3. Astrocytic Responses

Astrocytic responses following blast and NPLT were regionally heterogeneous but demonstrated several recurring patterns across anatomical locations. Most notably, GFAP percent staining tended to decrease following TBI and was frequently reduced further in NPLT + TBI, a pattern observed in the motor cortex, somatosensory cortex, corpus callosum, and thalamus. Some components of this pattern reached statistical significance, particularly for NPLT + TBI in the somatosensory cortex and corpus callosum, while similar trends were observed bilaterally and/or across multiple bregma levels. The hippocampus represented a notable exception to this broader pattern. Here, NPLT + Sham was associated with increased GFAP percent staining, including a significant increase at bregma −2.04, whereas staining was subsequently lower in TBI and NPLT + TBI. These findings indicate that neither blast nor NPLT produces a uniform change in GFAP staining throughout the brain and instead suggest substantial regional dependence in the astrocytic response.

GFAP+ cell density demonstrated a second recurring pattern that frequently differed from percent staining. TBI tended to increase GFAP+ cell density relative to Sham in the motor cortex and thalamus, whereas NPLT-treated groups tended to have lower cell densities in these regions. In contrast, the corpus callosum and hippocampus generally showed mildly reduced cell density following NPLT alone, followed by increases in TBI and/or NPLT + TBI. Although many individual comparisons did not reach significance, the recurrence of these trends across hemispheres and anatomical levels suggests that they may reflect biologically relevant regional responses that warrant further investigation.

An additional feature of these findings was that NPLT treatment alone produced region-specific alterations rather than consistently resembling untreated Sham animals. This observation parallels the microglial findings and is potentially relevant to the proposed preconditioning effect of NPLT. Rather than simply reversing blast-associated changes toward Sham values, NPLT + Sham appeared to establish its own regional cellular profile, while TBI produced a distinct pattern of alterations. Following blast, NPLT + TBI frequently exhibited a mixture of these treatment- and injury-associated patterns rather than consistently resembling either NPLT + Sham or TBI alone. Thus, one interpretation is that PrEP NPLT establishes an altered glial environment prior to injury that subsequently modifies the cellular response to blast. This interpretation is consistent with the broader preconditioning hypothesis proposed above; however, the functional significance of these astrocytic changes cannot be established from GFAP staining and cell density alone. Astrocyte morphology and molecular phenotype were not directly assessed and, therefore, these NPLT-associated alterations should not be interpreted as definitively protective or maladaptive.

### 3.4. Myelin Alterations

In contrast to the more variable glial responses, the myelin analysis demonstrated a localized blast-associated alteration that persisted despite NPLT. Overall, Solochrome staining in the corpus callosum did not significantly differ among treatment groups; however, at bregma 2.76, Solochrome staining was significantly decreased in both TBI and NPLT + TBI relative to Sham bilaterally. This bregma level is a medial location of the corpus callosum, which has been shown to be more vulnerable to TBI than other areas by Hartnell et al. [33]. That said, a single PrEP NPLT exposure was insufficient to prevent this localized reduction in myelin staining. The regional specificity of this effect is consistent with previous evidence that TBI produces nonuniform pathology along the corpus callosum, with the magnitude and time course of axonal alterations differing among the genu, mid-callosum, and splenium [34]. Regional differences in tissue composition may also influence NPLT effects. Gray and white matter exhibit distinct optical properties, particularly with respect to light scattering, and brain tissue displays spatial variation in acoustic attenuation and speed of sound [35]. This finding holds in studies investigating mono-modalities, including light waves in NIL therapy and acoustic waves in US therapy [36,37]. These tissue-dependent properties could contribute to regional differences in optical energy deposition and subsequent optoacoustic wave propagation following NPLT, although this possibility was not directly examined in the present study.

Considered together, the microglial, astrocytic, and myelin findings indicate that the cognitive benefit associated with PrEP NPLT does not require complete histological normalization at the endpoints examined here. This distinction is important because the therapeutic effect of NPLT may be better conceptualized as enhanced resilience to subsequent injury rather than complete prevention of tissue pathology. A primed neural environment could potentially preserve synaptic or network function in the presence of localized white matter and glial abnormalities. Alternatively, some pathological processes relevant to cognition may have occurred transiently at earlier timepoints and resolved before tissue collection. The current study cannot distinguish among these possibilities, but the combination of preserved cognition, persistent regional histopathology, and an NPLT-induced molecular response prior to injury provides a rationale for investigating functional and molecular measures alongside conventional histological endpoints in future prophylactic studies.

### 3.5. Limitations

There are limitations to this study. We evaluated pathology at a single post-injury timepoint, limiting the ability to determine whether inflammatory responses in regions such as the hippocampus and thalamus were transient, delayed, or persistent. Future longitudinal studies examining both earlier and later survival periods will be necessary to fully characterize the temporal evolution of NPLT-mediated neuroprotection after blast injury. Next, the study focused on male rats exclusively and did not include female rats. This results in a limitation to generalizability, as there is evidence of sex differences in TBI pathology [38]. Biological sex can influence TBI pathophysiology and recovery, and both preclinical and clinical studies have identified sex-dependent differences in outcomes following TBI. Importantly, however, the direction and magnitude of these differences vary according to injury model, injury severity, age, and the outcome examined, and, therefore, cannot be characterized as a uniformly protective or detrimental effect of sex. Future studies should include both males and females and be appropriately powered to evaluate sex as a biological variable.

Finally, the sample sizes for histological analyses were small (*n* = 4). While this is a common occurrence in pathohistological assessment, this study required a large number of statistical comparisons between parameters. A small sample requiring many comparisons limits the strength of our conclusions. Multiple comparisons were accounted for by post hoc statistical correction; however, it is important to acknowledge the limitation a small sample size has on the power of the analyses.

## 4. Methods

### 4.1. Animals

All animals were adult male Sprague-Dawley rats (Charles River Laboratories, Wilmington, MA, USA) ranging in weight from 250 to 350 g. Animal care and experimental use was approved (Protocol No. 0910062) and under the guidance of the Institutional Animal Care and Use Committee (IACUC) of the University of Texas Medical Branch, Galveston, Texas, and in accordance with the National Institutes of Health Guide for the Care and Use of Laboratory Animals. All animals were given food and water ad libitum and were housed in a controlled environment animal housing facility.

### 4.2. NPLT

To receive NPLT treatment, all rats were anesthetized using 3% isoflurane delivered through a nose cone and their heads were shaved. Upon lack of paw reflex, NPLT was administered transcranially for a duration of 5 min through a 3 mm-diameter fiber optic bundle system at a location 2.5 mm parasagittal from the midline, approximately −2.5 bregma. Treatment was provided bilaterally for Study 1 and unilaterally for Study 2. The fiber optic bundle system was held in place using a stereotaxic holder. The optical parametric oscillator generated short (10 ns) pulses of near-infrared light (808 nm) with an incident energy of 5 mJ and a pulse repetition rate of 20 Hz. Total dose for the 5 min treatment was 300 J/cm^2^. The same protocol was applied to rats assigned to Sham treatment up to placing them on the stereotaxic platform, but Sham rats were not exposed to NPLT.

### 4.3. Study 1

#### 4.3.1. Experimental Design

Naive animals (*n* = 7) were randomly assigned to receive Sham (*n* = 4) or bilateral NPLT treatment (*n* = 3) and were euthanized 24 h later. To ensure data integrity, objectivity, and strict scientific rigor, treatment groups in all analyses were blinded to all investigators except for the Primary Investigator. The study was reported in accordance with the ARRIVE guidelines.

#### 4.3.2. Tissue Processing and qRT-PCR

In Study 1, rats were euthanized twenty-four hours after treatment. Rats were anesthetized using 4% isoflurane and transcardially perfused with saline. The brains were removed, and the hippocampus was rapidly frozen on dry ice and stored at −80 °C.

#### 4.3.3. RNA Isolation, Reverse Transcription, and Quantitative Real-Time PCR

Total RNA was isolated from hippocampal tissue using the RNA Aqueous kit (Ambion/Thermo Scientific, Waltham, MA, USA) according to the manufacturer’s protocol and then D-Nase treated at 37 °C for 20 min to remove any traces of genomic DNA. The concentration of the total RNA was measured with the Nano-Drop One spectrophotometer (Thermo Scientific, Waltham, MA, USA). Then, 200 ng of Total RNA was reverse transcribed using the High-Capacity cDNA Reverse Transcription Kit (Applied Biosystems/Thermo-Scientific, Waltham, MA, USA) according to the manufacturer’s protocol. Quantitative Real-Time PCR was performed on a Light Cycler 96 instrument (Roche, Indianapolis, IN, USA) with Taqman chemistry probes (Applied Biosystems/Thermo-Scientific, Waltham, MA, USA) to measure gene expression levels of BDNF and GAPDH using the following thermal profile: 50 °C for 2 min (1 cycle), 95 °C for 2 min (1 cycle), and 95 °C for 15 s/60 °C for 1 min (40 cycles). Each PCR reaction was performed in triplicate. Normalization to the housekeeping gene GAPDH was performed by calculating the ΔCt for each gene of interest (GOI) by subtracting the Ct value of gene of interest from the Ct value of GAPDH. All data from the PCR were collected and analyzed using the Light Cycler 96 software version 1.1.0.1320. Data analysis was performed using the ΔΔCt method with NPLT compared to relative Sham levels.

### 4.4. Study 2

#### 4.4.1. Experimental Design

The experimental timeline is shown in Figure 1. Animals were randomly assigned to receive Sham injury or bTBI and then further randomized to receive NPLT or Sham PrEP treatment 24 h prior to injury. On post-injury days (PIDs) 1–5, the animals underwent neurological and vestibulomotor assessment with the Neuroscore, beam balance, and beam walk tests. On PIDs 13–17, the animals were subjected to the Morris Water Maze (MWM) test to assess cognitive behavior. On PID 17, all animals were sacrificed. Twelve rats per treatment group were utilized for behavior analyses. Four rats per group were perfused-fixed for histological analyses and brains of eight rats per group were removed and fresh frozen for future study. To ensure data integrity, objectivity, and strict scientific rigor, treatment groups in all analyses were blinded to all investigators except for the Primary Investigator. The study was reported in accordance with the ARRIVE guidelines.

#### 4.4.2. Blast Injury

Animals were anesthetized (4% isoflurane) using a nose cone and ventilated (2% isoflurane) in 80:20 O_2_:room air using a small animal ventilator (Harvard Apparatus, Inc., Holliston, MA, USA). Animal core body temperature was measured and monitored using a rectal telethermometer (Thermalert Monitoring Thermometer, Physitemp Instrument, Inc., Clifton, NJ, USA) and temperature was maintained at 30–35 degrees Celsius using a thermostatically controlled water blanket (Mul-T-Pad Temperature Therapy Pad, Gaymar Industries, Inc., Orchard Park, NY, USA). The anesthetized animal was then placed on the specimen tray and secured using Velcro^®^ straps in a transverse prone position with the head supported by a leather sling, ensuring the head was perpendicular to the shock wave. Foam heads were placed into the ears, and the nose cone was removed. The specimen tray with the animal was secured into the advanced blast simulator and only the head was inserted into the expansion chamber. At the return of a withdrawal reflex to paw pinch, the rat was subjected to bTBI (20.9 psi ±1.14, 138 kPa ±7.9) or Sham bTBI. Shock wave overpressures had a mean rise time of 0.37 milliseconds ± 0.006 and a duration of 3.50 milliseconds ± 0.063, calculated suing the slope and y-intercept formula. After the blast was delivered, the animal was immediately removed from the chamber and placed supine. Sham bTBI animals were anesthetized and secured in the same manner but were not subjected to bTBI. All animals were subjected to the righting reflex (RR) test immediately following removal from the chamber and the time taken to roll over from supine to an upright position was noted.

#### 4.4.3. Vestibulomotor Function Tests

To test the ability of NPLT to ameliorate bTBI-induced neurological dysfunction, neurological reflexes, gross vestibulomotor function, and fine motor coordination were measured via a composite Neuroscore, beam balance, and beam walk, respectively. Rats were trained in each task on PIDs –3 and –2, tested on PID –1 for baseline measurement, and tested again on PIDs 1–5.

##### Neuroscore

Below is an ordered list of the reflex tests performed to assess the neurological function of each animal. The characteristics of normal and abnormal reflex responses are detailed by Hausser et al. [17]. Normal responses were scored 0 and abnormal were scored 1. Each test was repeated three times in a single sitting for a potential score of 21 (7 × 3 = 21). The higher the score, the greater the neurological dysfunction:

1. Forelimb Flexion Test (0–1)
2. Hind limb Flexion Test (0–1)
3. Visually Triggered Placing Test (0–1)
4. Contact Triggered Placing Test, right (0–1)
5. Contact Triggered Placing Test, left (0–1)
6. Hind Paw Grasping Reflex Test, right (0–1)
7. Hind Paw Grasping Reflex Test, left (0–1)

##### Beam Balance

A wooden beam measuring 60 × 1.75 × 4 cm^3^ was raised 90 cm off the floor and surrounded by a padded safety box. One side includes a 30 × 30 cm^2^ barrier. In a 60 s trial, animals were pre-trained to balance on the beam. Over three consecutive 1 min trials, animals were scored on an ordinal scale, with (1) being normal, steady balance, and (6) being falls with no attempt to balance. Scores were averaged by day.

##### Beam Walk

A wooden beam measuring 100 × 2.5 × 4 cm^3^ was raised 1 m off the floor. Pegs measuring 2 cm in height were placed equidistantly along the beam as obstacles and a darkened goal box (28 × 18 × 18 cm^3^) was attached at one end. A lamp and white noise machine were placed at the starting point. In a 60 s trial, animals were pre-trained to traverse the beam and tested over three consecutive 1 min trials. Time to traverse the beam was averaged by day.

#### 4.4.4. Working Memory and Spatial Learning Test

To test the ability of NPLT to ameliorate bTBI-induced cognitive dysfunction, rats underwent the Morris Water Maze (MWM) on PIDs 13–17 according to the protocol described by Sell et al. [39]. The animals were placed into a pool tank 6 ft in diameter and 2.5 ft deep with the water level 2 cm above hidden safety platforms. Animals were randomly assigned to one of four starting locations in the pool with a hidden platform nearby for paired trials. In Trial 1 (T1), the animal was placed in the water at the starting location and allowed 120 s to locate the hidden platform. When the animal located the platform, they were allowed 15 s to rest, then were placed at the same starting location for Trial 2 (T2) and provided 120 s to locate the same platform. Between each of four sets of paired trials, corresponding to the four different starting locations, the animals rested in heated enclosures for 4 min. Over five consecutive days, all animals received four pairs of trials daily. The swim path of the animals was recorded and tracked using a ceiling-mounted camera and the tracking software Anymaze version 6.3 (Anymaze, Stoelting Co., Wood Dale, IL, USA). Time-to-platform was averaged for T1 and T2, and T2 latency to locate the platform represents a measurement of working memory.

#### 4.4.5. Tissue Processing and Immunostaining

In Study 2, rats were euthanized on PID 17 after completion of the MWM test. Rats were anesthetized and perfused with saline followed by freshly prepared phosphate-buffered formaldehyde solution (pH 7.4). The brains were dissected and post-fixed in formaldehyde for 12–16 h at room temperature, transferred to a phosphate-buffered solution (PBS), and shipped to Neuro-Science Associates (NSA, Knoxville, TN, USA), where a patented MultiBrain^®^ technology was used to process 16 brains at once. To do so, the samples were treated overnight using 20% glycerol and 2% dimenthylsulfide. All 16 brains were then embedded in a gelatin matrix (MultiBrain^®^ technology). The gelatin block containing the brains was then cured in 2-mehtylbutane and chilled on crushed dry ice. Once frozen, the block was mounted to an AO 860 sliding microtome and coronally sectioned at 40 µm. The sections were collected into a series of 24 cups containing antigen preserve solution (50% PBS pH 7.0, cat# 70383, Sigma-Aldrich, St Louis, MO, USA/50% Ethylene Glycol, cat# 107-21-1, Alfa Aesar, Ward Hill, MA, USA/1% Polyvinyl Pyrrolidone, cat# 9003-39-8, Fisher Scientific, Fair Lawn, NJ, USA). The sections were mounted to glass sides and processed for immunohistological staining of Iba1 (to identify microglia), GFAP (to identify astrocytes), and Solochrome cyanine (to identify intact myelination).

#### 4.4.6. Image Acquisition and Quantification

The images were acquired using a BZ-X710 microscope (Keyence America, Itasca, IL, USA) at 10× zoom using the BZ-X analyzer software version 1.4.0.1 (Keyence America, Itasca, IL, USA). The Paxino and Watson rat brain atlas was used to determine the boundaries of the motor cortex, somatosensory cortex, corpus callosum, hippocampus, and thalamus. Within each, quantification the percent of area stained (Iba1, GFAP, and Solochrome), cellular count (GFAP and Iba1), and transformation index (Iba1) was determined by using the ImageJ-Fiji software (https://imagej.net/software/fiji/ (accessed on 10 August 2026)) (National Institutes of Health, Bethesda, MD, USA) for the bregma levels.

### 4.5. Statistical Analysis

Data are expressed as mean ± standard error of the mean (SEM). A 95% confidence interval was assumed (α = 0.05), and analyses were performed using GraphPad 10 Prism software. Significant differences were noted when *p* < 0.05. Power analysis, based on preliminary data with an effect size of 0.20, indicated a need for 12 animals per treatment group to achieve 80% power to detect a significant difference between injury and NPLT using a two-way ANOVA with a 0.05 level of significance. Factors were injury (Sham and TBI) and treatment (NPLT and no treatment). For histology, based on preliminary data with an effect size of 0.30, power analysis indicated a need for 6 animals per treatment group to achieve 80% power to detect a significant difference between injury and NPLT using a two-way ANOVA with a 0.05 level of significance. Note that group sample sizes were satisfied for behavior; however, a mechanical breakdown of a laboratory refrigerator during a weather event led to the loss of samples and only 4 animals per treatment group were achieved for histology.

In Study 1, data were normalized to a housekeeping control, in this case glyceraldehyde 3-phosphate dehydrogenase (GAPDH). Treatment groups were compared with Student’s *t*-test. In Study 2, for the beam balance, beam walk, and Neuroscore, a 4 (treatment group: Sham, NPLT + Sham, TBI, and NPLT + TBI) × 5 (PIDs 1–5) two-way analysis of variance (ANOVA) was performed, followed by Tukey’s post hoc comparisons. For the MWM test, mean latency ± SEM of T1–T2 for each group for PIDs 13–17 were analyzed with two-way ANOVA followed by Tukey’s post hoc comparisons. Righting reflex latency by treatment group was compared using Brown–Forsythe and Welch ANOVA. For the histological data, mean percent stain per area and cell count per area were compared by treatment group via one-way ANOVA followed by Tukey’s HSD comparisons and by two-way ANOVA with Tukey’s HSD test to stratify treatment groups by bregma level.

## 5. Conclusions

In this study, we demonstrated that a single pre-exposure application of NPLT provides partial protection against blast-induced neurotrauma in a rat model of mild blast TBI. A single PrEP NPLT treatment administered 24 h prior to mild blast was associated with preservation of cognitive performance and more modest attenuation of fine motor dysfunction. These functional benefits occurred despite persistent localized myelin abnormalities and heterogeneous glial responses, indicating that NPLT does not uniformly prevent blast-associated tissue pathology. Instead, the histological findings suggest regionally patterned modulation of microglial and astrocytic responses. Importantly, increased hippocampal BDNF 24 h after NPLT in naive rats demonstrates that a single treatment induces a sustained molecular response before injury, while complementary findings from our laboratory demonstrate that NPLT can produce persistent cellular modifications associated with resilience to a subsequent toxic challenge. Together, these findings support a model in which PrEP NPLT may establish a preconditioned neural state that promotes functional resilience following blast. Determining whether this resilience results from enhanced cellular stress responses, synaptic plasticity, preservation or compensation of neural network function, or a combination of these mechanisms represents an important direction for future investigation. Additional future studies are warranted to further develop optimal dosing and timing to determine whether repeated pre-injury NPLT administration can further attenuate blast TBI histopathology.

## Data Availability

Study data are available upon request sent to the corresponding author.
